# Multi-trait selection of tropical maize hybrids for combined productivity, agronomic stability, and aflatoxin resistance

**DOI:** 10.3389/fpls.2026.1964715

**Published:** 2026-09-30

**Authors:** Victor Adelodun Diekade, Ayodeji Abe, Mamadou Mboup, Silvestro Meseka, Wende Mengesha, Abebe Menkir, Melaku Gedil, Alejandro Ortega-Beltran, John Derera

**Affiliations:** 1 Department of Crop and Horticultural Sciences, Faculty of Agriculture, University of Ibadan, Ibadan, Nigeria; 2 International Institute of Tropical Agriculture (IITA), Ibadan, Nigeria; 3 Plant Biology Department, Cheikh Anta Diop University, Dakar-Fann, Senegal

**Keywords:** aflatoxin contamination, Aspergillus ear rot, grain yield, multi-trait genotype-ideotype distance index, multi-trait selection, tropical maize hybrids

## Abstract

Aspergillus ear rot (ear rot) and aflatoxin contamination, caused mainly by *Aspergillus flavus*, threaten maize productivity, grain quality, and food safety in tropical and sub-tropical regions. Although significant breeding progress has been made, selection for resistance remains challenging due to environmental conditions, genotype × environment interaction (G×E), low heritability of resistance traits, and the need to simultaneously improve productivity. The objectives of this study were to: assess the effect of genotype, environment and G×E, examine trait relationships, and identify superior maize hybrids with favourable multi-trait performance. Eighty-three maize inbred lines were crossed with two testers using a line × tester mating design. The resultant 166 testcrosses and six checks were evaluated across four environments with two replications in each environment under artificial inoculation using a highly toxigenic *A. flavus* isolate (La3228). Data were analysed using linear mixed models, with variance components estimated by REML. Environmental effects were significant for aflatoxin accumulation and ear rot (*p* ≤ 0.05), while hybrids differed significantly for grain yield and ear rot (*p* ≤ 0.01). There was no significant G×E interaction for grain yield, aflatoxin accumulation and ear rot (*p* ≥ 0.05). Broad-sense heritability estimate was high for grain yield (H^2^ = 0.62), moderate for ear rot (H^2^ = 0.36) and low for aflatoxin (H^2^ = 0.22), indicating strong environmental influence on the expression of the resistance-related traits. Grain yield was not significantly correlated with aflatoxin accumulation (r = 0.08), suggesting that selection for grain yield may be independent of aflatoxin accumulation. However, grain yield was significantly and negatively correlated with ear rot (r = -0.20*). The first two principal components of the genotype plus genotype × environment (GGE) biplot explained 76.4% of the total variation among the selected hybrids and identified two mega-environments, revealing differential adaptation among hybrids. The sequential application of multi-trait genotype–ideotype distance index (MGIDI) and GGE biplot identified five testcrosses, En80, En74, En102, En157, and En65 combining competitive grain yield, favourable stability, and resistance-related traits. These hybrids are promising for further multi-environment evaluation for their potential contribution to the development of maize hybrids with improved productivity and reduced aflatoxin accumulation in tropical and sub-tropical regions.

## 1 Introduction

Maize (*Zea mays* L.) is one of the most important cereal crops globally. It serves as a major source of food, an essential component of livestock feed, and a key raw material for industries. Together with wheat and rice, maize forms the backbone of global cereal production systems that sustain food security for billions. Its average global yield and total production stands at approximately 5.8 t/ha and 1.22 billion tonnes, respectively, surpassing that of rice and wheat (FAOSTAT, 2024). However, despite its importance and high productivity potential, substantial yield disparities persist between developed and developing regions.

Despite being the most widely cultivated cereal in Africa, maize productivity remains low, with an estimated average yield of 2.07 t/ha (FAOSTAT, 2024). This low productivity and wide yield gap are driven by several constraints including, limited access to improved varieties (Aramburu-Merlos et al., 2024), poor soil fertility (Okunlola et al., 2023), drought stress (Menkir et al., 2024), and biotic stresses such as infestation of striga (Oyekale and Badu-Apraku, 2025), fall army worm (Abubakar et al., 2025), and infection of fungal diseases and their associated mycotoxins (Diekade et al., 2026). Biotic stresses alone account for substantial grain yield losses in maize, with estimates ranging from 30 to 100% depending on stress type, severity level, and environmental conditions (Njeru et al., 2023).

Aspergillus ear rot (ear rot), caused mainly by *Aspergillus flavus*, and subsequent aflatoxin production continue to threaten maize productivity and compromise its safety (Logrieco et al., 2021). This problem is particularly severe in sub-Saharan Africa, where maize is a staple food for over 300 million people and a major source of daily calorie intake (Badu-Apraku and Fakorede, 2017). Aflatoxins are toxic secondary metabolites produced by several *Aspergillus* species, and with the ability to cause cancer, suppress immune system, cause birth defect, damage liver in both humans and animals, which are sometimes fatal (JECFA, 2018; Frisvad et al., 2019). Beyond being a global health concern, aflatoxin contamination of maize causes significant economic losses of up to US$163 million annually in the USA, and US$750 million in Africa (Wu, 2015; Gbashi et al., 2018; Mesterházy et al., 2024). The impact of aflatoxin contamination of maize is further exacerbated in developing countries due to limited capacity to monitor its occurrence coupled with the international trade restrictions on contaminated grains, which hinder farmers from getting premium earnings from the sale of their produce (Widstrom, 1996; Matumba et al., 2017; Goda et al., 2025). Given the foregoing, there is an urgent need for a sustainable approach to mitigate aflatoxin contamination of maize grain.

Prevalence of conducive environmental conditions such as high temperature and drought stress favours the growth of *A. flavus*, its colonization of maize grains, and subsequent aflatoxin production (Bunny et al., 2024; Casu et al., 2024; Bereziartua et al., 2025). In addition, injuries caused by insect pests, mechanical damage during harvesting, poor harvest and drying practices, threshing, and poor post-harvest handling amplify aflatoxin accumulation (Ab Dullah et al., 2026; Kaburi et al., 2026; Syraji et al., 2025). In tropical and sub-tropical regions, high temperature, heat, and drought stresses, particularly during grain filling and maturation stages, lead to pronounced variation in aflatoxin contamination across locations and seasons (Bereziartua et al., 2025; Yohannis et al., 2025). Such environmental variation can influence the expression of aflatoxin accumulation and other resistance-related traits, highlighting the importance of multi-environment evaluation. Multi-environment evaluation of genetic materials provides a viable approach to accurately assess host resistance and identify maize hybrids with stable performance across contrasting environments. Several analytical approaches have been developed to investigate genotype performance across environments. Among these, the genotype plus genotype × environment (GGE) biplot has been widely employed to visualize genotype performance and stability, identify mega-environments, and facilitate the selection of superior and widely adapted genotypes (Yan and Rajcan, 2002; Yan and Kang, 2003; Yan and Tinker, 2006).

Several approaches, including good agricultural practices (GAP) and use of biocontrol agents have been explored in the management of aflatoxin contamination. However, breeding for resistance remain a valuable and cost-effective component of integrated aflatoxin management approach (Guo et al., 2017; Khadgi et al., 2025; Manzoor et al., 2026). In that regard, considerable breeding progress has been made, including the identification of resistant germplasm (Menkir et al., 2008; Scully et al., 2016; Mboup et al., 2024; Mollel et al., 2025; Diekade et al., 2026), and identification aflatoxin-resistant quantitative trait loci (QTLs) (Warburton et al., 2015; Dhakal et al., 2016; Smith et al., 2019; Ogunola et al., 2021; Li et al., 2025). However, resistance to aflatoxin contamination is a complex polygenic trait with strong environmental influence and low heritability, which limits selection efficiency (Warburton and Williams, 2014; Soni et al., 2020). Additionally, most of the identified resistance sources are lacking in desirable agronomic traits such as high grain yield, which hinder their direct use in breeding programmes (Rose et al., 2017; Mollel et al., 2025). These challenges present the need for integrated breeding approaches that simultaneously improve grain yield, resistance to aflatoxin accumulation and stability across diverse growing environments. Moreover, the evaluation of hybrids carrying introgressed resistance QTL under diverse tropical environments is necessary to validate the effectiveness of resistance sources and identify promising hybrids for deployment.

Genetic variability among maize genotypes for resistance to ear rot and aflatoxin accumulation has been reported previously (Okoth et al., 2017; Garrido-Bazan et al., 2018; Meseka et al., 2018). However, there is a dearth of information on the performance, stability, and multi-trait superiority of tropical maize hybrids derived from parental lines carrying aflatoxin resistance QTL across multiple environments. In addition, information on hybrids that combine high and stable yield with resistance to ear rot and aflatoxin under tropical conditions using modern multi-trait selection tools is limited. Among the recently developed multi-trait selection approaches, the Multi-trait Genotype–Ideotype Distance Index (MGIDI) has gained considerable attention because it integrates multiple traits into a single selection criterion without requiring subjective economic weights (Mau et al., 2019; Liu and Qin, 2021). The method combines factor analysis with ideotype-based selection to reduce dimensionality, minimize multicollinearity among traits, and identify genotypes with balanced overall performance while also revealing their strengths and weaknesses for individual trait groups (Olivoto and Nardino, 2021; Pallavi et al., 2024).

In the present study, the effects of genotype, environment, and G×E on ear rot, aflatoxin accumulation, and grain yield were investigated in tropical maize hybrids. In addition, the relationships among ear rot, aflatoxin accumulation, and grain yield were examined to better understand trait interdependence under field conditions. The extent to which G×E influences the identification of high-performing hybrids was also assessed. Furthermore, the effectiveness of integrating genotype plus genotype × environment (GGE) biplot and multi-trait genotype–ideotype distance index (MGIDI) approaches for simultaneous selection of hybrids based on productivity, stability, and resistance-related traits was evaluated.

## 2 Materials and methods

### 2.1 Genetic materials and field evaluations

Eighty-three advanced yellow-kernel inbred lines carrying aflatoxin-resistant QTLs and two testers belonging to complementary heterotic groups were used in this study. The testers comprised one aflatoxin-resistant and one aflatoxin-susceptible inbred line. The 83 lines were developed from a backcross involving the temperate line Mp715 as the donor of aflatoxin-resistant QTLs and the elite line IITATZI1394 as the recurrent parent by the Maize Improvement Program of the International Institute of Tropical Agriculture (IITA), Ibadan. Following the backcross, there were three cycles of marker-assisted enrichment with selection for plants carrying markers linked to the QTLs. After the third cycle, self-pollinated plants derived from the backcross were selected for desirable agronomic and adaptive traits. These were planted ear-to-row in the following season. The 83 advanced lines were then developed through five subsequent generations of selfing with selection for desired agronomic attributes and resistance to major foliar diseases. The 83 lines were crossed with the two testers using a line × tester mating design during the dry season of 2023 at the IITA research field in Ibadan, Nigeria, resulting in 166 testcross hybrids.

The 166 testcrosses and six checks totaling 172 entries (**Supplementary Table 1**) were evaluated at two locations namely: Ibadan (7°30′ N, 3°54′ E, 190 m above sea level) and Ikenne (3° 42′ E, 6° 54′ N, 30 m above sea level) in Nigeria during the 2024 and 2025 growing seasons. In this study, environments were defined by the combination of location and growing season (location × year matrix), resulting in four environments: Ibadan 2024 (IB24), Ibadan 2025 (IB25), Ikenne 2024 (IK24), and Ikenne 2025 (IK25). The two experimental locations belong to different agroecological zones of Nigeria and characterized by different altitude, temperature range, and rainfall pattern as shown in **Table 1**. The six checks comprised of a hybrid involving a cross between the two testers (T1×T2), two genetic check hybrids (8425–8 and 8338-1), and three commercial hybrids (Ife hybrid 3, Ife hybrid 4, and SC645). The T1×T2 was included as a breeding reference check for comparison with the derived testcross hybrids. The two genetic check hybrids represented advanced breeding materials in the IITA breeding programme and served as tolerant and susceptible checks, respectively, providing contrasting resistance benchmarks. The three commercial check hybrids were included as standard production checks to benchmark the performance of the testcross hybrids under the target production environments. The experiment was laid as a 43 × 4 alpha lattice design with two replications. Each entry was planted to single row plots which are 4.0 m long, with inter- and intra-row spacing of 0.75 m and 0.5 m, respectively. Two border rows and guard rows were planted around the experimental plots. Three seeds were sown per hill and seedlings thinned to two at three weeks after sowing. Standard agronomic practices such as weeding and fertilizer application were followed at each location.

**Table 1:** Agroecological characteristics and weather conditions of the experimental environments (location × year) used in Nigeria.

Location	Year	Environment code	Agroecological zone	GPS Coordinates	Altitude (M asl)	T_min_ (˚C)	T_max_ (˚C)	Total rainfall (mm)	RH (%)
Ibadan (IB)	2024	IB24	Derived savanna	7°30′ N, 3°54′ E	190	22.94	29.77	693.01	81.01
Ikenne (IK)	2024	IK24	Rainforest	6° 54′ N, 3° 42′ E	30	23.29	30.13	1, 019.00	93.57
Ibadan (IB)	2025	IB25	Derived savanna	7°30′ N, 3°54′ E	190	22.80	29.50	632.80	83.00
Ikenne (IK)	2025	IK25	Rainforest	6° 54′ N, 3° 42′ E	30	23.00	28.10	941.40	98.40

M asl, Meter above sea level; T_min_, Minimum temperature; T_max_, Maximum temperature; RH, Relative humidity.

### 2.2 Inoculum preparation and field inoculation

A highly toxigenic *A. flavus* isolate, La3228, belonging to fungal collection of IITA Pathology and Mycotoxin/Aflasafe Unit was used in the current study. The isolate is known for its well-characterized virulence and consistency of producing high concentration of aflatoxins (B1 and B2) and has been used in several aflatoxin resistance studies in maize (Meseka et al., 2018; Bankole et al., 2022; Mboup et al., 2024; Diekade et al., 2026). Inoculum was prepared by growing La3228 on 50 g sterilized sorghum grains in 250 mL conical flasks. The flasks were incubated at 31°C in the dark and shaken once daily for the first few days to allow even distribution of the fungus on the grains and to prevent clumping. After three weeks of incubation, conidia were washed from the grains using sterile TWEEN^®^20 solution (Thermo Fisher Scientific, Waltham, MA, USA) at 20 drops/L of sterile distilled water and filtered through four layers of sterile cheesecloth. The concentration of the inoculum was determined using hemocytometer and final concentration adjusted to 8 × 10^6^ conidia/mL. The conidia suspension was refrigerated for no more than 48 hours at 4°C before field inoculation. Top ears of seven plants per plot were inoculated 8–10 days after mid-silking using the silk-channel inoculation method (Zummo and Scott, 1989). The ears were inoculated by slowly dispensing 3 mL fungal suspension using 10-ml syringe-pipette and 18-gauge hypodermic needle. The inoculated ears were tagged and covered with pollination bags. Some randomly selected non-inoculated ears were also covered to serve as control.

### 2.3 Agronomic data collection

Data were collected on days to anthesis, days to silking, ear aspect, plant height, ear height, field weight and grain yield. Days to anthesis and days to silking were recorded as the number of days from planting to when 50% of plants in a plot had shed pollens and silk emerged, respectively. Anthesis-silking interval was computed as the difference between days to silking and days to anthesis. Plant height and ear height were measured in cm as the distance from the base of the plant to first tassel branch and the node bearing the top ear, respectively. Ear aspect was scored on a scale of 1 to 5 with 1 representing clean and well filled ears, and 5 representing ears with scanty and rotten or damaged kernels (Iseghohi et al., 2020). At harvest, field weight was measured in kg as the weight of the maize cobs per plot after de-husking. Grain moisture content was determined using a portable Kett PM-450 grain moisture tester (Kett Electric Laboratory Co., Ltd., Japan). Grain yield was calculated from the field weight and adjusted to 15% moisture content, assuming 80% shelling percentage. The formula for calculating grain yield is given in **Equation 1** (Ngoune Tandzi and Mutengwa, 2020) as:

(1) $$GY(t\;ha^{-1})=\frac{FWT\times 10\times (100-MC)\times 0.8}{(100-adjusted\;MC)\times plot\;area}$$

Where GY is grain yield measured in tonnes per hectare, FWT is field weight in kg, and MC is moisture content.

### 2.4 Assessment of Aspergillus ear rot

At physiological maturity, inoculated ears in each plot were harvested by hand at 12-18% moisture content. The percentage of ear rot symptoms on each ear was estimated visually following a method adapted from Henry et al. (2009). Aspergillus ear rots were rated on a scale of 1 to 7, where 1 = 0% rotten kernels, 2 = 1-3% rot, 3 = 4-10% rot, 4 = 11-25% rot, 5 = 26-50% rot, 6 = 51-75% rot, and 7 = 76-100% rot. Thereafter, ears from each plot were bulked, hand-shelled and properly mixed before taking a representative sample of 250 g. The sample was milled using a laboratory blender (Warring Commercial, Torrington, CT, USA) and preserved at 4°C until the extraction of aflatoxin was performed.

### 2.5 Aflatoxin extraction and quantification

Aflatoxin was extracted and quantified from a homogenized 20 g sub-sample taken from the 250 g milled sample from each plot as previously described (Ortega-Beltran et al., 2014). Briefly, 4 μL of the dissolved dichloromethane extract of each entry was directly spotted alongside aflatoxin standards (Supelco^®^, Bellefonte, PA, USA) on thin-layer chromatography plates (silica gel 60, EMD Millipore, Darmstadt, Germany) and developed with diethyl ether-methanol-water (96:3:1) solution. After drying, the plates were visualized under UV light for the presence or absence of aflatoxins. Positive results showed blue fluorescence, while negative results had no fluorescence. The plates were then read with a CAMAG TLC 4 Scanner densitometer coupled with VisionCats 3.1 (CAMAG AC, Muttenz, Switzerland) to quantify the aflatoxins.

### 2.6 Statistical analysis

Data on aflatoxin concentration was transformed using log(x+1), after assessment for normality with Shapiro-Wilk test in SAS version 9.4 (SAS Institute Inc., 2013). Following model fitting to the transformed data, residual normality was assessed using the Shapiro–Wilk test and Q–Q plots. A combined Analysis of Variance (ANOVA) was done for all data across environments using the PROC MIXED procedure in SAS version 9.4 (SAS Institute Inc., 2013). In the combined analysis, environment was treated as fixed effect, while genotype, G×E, replication, block, and residual effects were considered as random effects. For each trait, the following mixed model (Equation 2) was fitted:

(2) $$Y_{ijkl}=\mu +E_{i}+R_{j(i)}+B_{k(ij)}+G_{l}+{\left(GE\right)}_{il}+\epsilon_{ijkl}$$

​where $Y_{ijkl}$​ is the observed value of the $l^{th}$ genotype in the $k^{th}$block of the $j^{th}$ replication within the $i^{th}$ environment; μ is the overall mean; $E_{i}$​, $R_{j(i)}$​, and $B_{k(ij)}$ represent the effects of environment, replication, and block, respectively; $G_{l}$ is the genotypic main effect; ${\left(GE\right)}_{il}$ is the genotype × environment interaction effects; and $\epsilon_{ijkl}$ is the residual error. The alpha-lattice structure was accounted for through replication and block effects within environments; no additional spatial adjustment or residual covariance structure was applied. Significantly different means among the environments were separated using Tukey’s honestly significant difference (HSD) test at 5% significance level. Variance components for genotype, G×E, and residual effects were estimated using the Restricted Maximum Likelihood (REML) method implemented in PROC MIXED of SAS. The estimates were then used to calculate the percentage contribution of each source of variation to the total phenotypic variance. Broad-sense heritability across environments was estimated (Equation 3) and classified as low (<30%), moderate (30-60%), and high (>60%) (Johnson et al., 1955):

(3) $$H^{2}=\frac{\sigma_{g}^{2}}{\sigma_{g}^{2}+\frac{\sigma_{ge}^{2}}{n_{E}}+\frac{\sigma_{e}^{2}}{n_{E}n_{R}}}$$

where: $\sigma_{g}^{2}$ is the genotypic variance $,\sigma_{ge}^{2}$ is the G×E variance, $\sigma_{e}^{2}$​ is the residual variance, $n_{E}$​ is the number of environments, and $n_{R}$ is the number of replications. Best linear unbiased predictions (BLUPs) for genotypes were extracted from the combined ANOVA fitted as linear mixed models in R using the lme4 package (R Core Team, 2025) and were used for all downstream multivariate analyses, including Pearson correlation analysis, MGIDI, and GGE biplot analysis. Pearson correlation analysis was performed to assess the pairwise associations among the evaluated traits, and the resulting correlation matrix was visualized using the corrplot package in R. Spearman’s rank correlation analysis was performed to assess the associations among MGIDI, grain yield, and aflatoxin accumulation. Genotype selection across multiple traits was performed using the Multi-trait Genotype–Ideotype Distance Index (MGIDI) proposed by Olivoto and Lúcio (2021), with the R package metan. Traits were rescaled so that all traits range from 0 to 100 using the following (Equation 4);

(4) $$rX_{ij}=\frac{\eta_{nj}-\phi_{nj}}{\eta_{oj}-\phi_{oj}}(\theta_{ij}-\eta_{oj})+\eta_{nj}$$

Where η*_nj_* and φ*_nj_* denotes the new maximum and minimum values for trait j, respectively, after rescaling, η_oj_ and φ_oj_ are the original maximum and minimum values for trait j. θ_ij_ is the original value of the j-th trait for the i-th genotype.

In the case of traits such as days to anthesis, days to silking, aflatoxin accumulation, Aspergillus ear rot, and ear aspect, where lower values are desired, η*_nj_* is set to 0 and φ*_nj_* to 100. Whereas, for traits like grain yield, ear height, and plant height, where higher values are preferred, η*_nj_* is set to 100 and φ*_nj_* to 0. The ideal genotype was represented by a value of 100 for all evaluated traits after rescaling.

Thereafter, rescaled data were subjected to factor analysis to reduce dimensionality and account for correlation among the traits as described in Equation 5;

(5) X =μ+ LF + ϵ

X is a 1 ×p vector of rescaled observations, while μ is 1 ×p vector of standardized means. L is a p×f matrix of factorial loadings. The vector *f* is a  1×p vector that represents the common factors, and ϵ is a 1 ×p vector of residuals. Here, *p* and *f* denote the number of retained traits and the number of common factors, respectively. Eigenvalues and eigenvectors were derived from the correlation matrix of $rX_{ij}$​, and only factors with eigenvalues greater than 1 were retained. The scores were computed as shown in Equation 6:

(6) $$F=Z{\left(A^{T}R^{-1}\right)}^{T}$$

Where F is a g ×f matrix of factor scores, Z is a g ×p matrix containing the standardized trait means, A is a p ×fmatrix of canonical loadings, and R is a p ×p correlation matrix among the analyzed traits. Here, *g*, *f*, and *p* denote the number of accessions, retained factors, and evaluated traits, respectively. Subsequently, the MGIDI index was computed as the Euclidean distance between each genotype and the ideotype in the factor space as shown in Equation 7:

(7) $$MGIDI_{i}={\left[\sum_{j=1}^{f}{\left(Y_{ij}-Y_{j}\right)}^{2}\right]}^{1/2}$$

Where $Y_{ij}$ is the score of the $i^{th}\;$genotype on the $j^{th}$ factor (i =1, 2, …, g; j = 1, 2, …, f), with g and f representing the number of testcross hybrids and retained factors, respectively. $Y_{j}$ denotes the score of the ideotype on the $j^{th}$ factor.

The genotype with the lowest MGIDI value was considered closest to the ideal genotype, indicating more desirable performance across all evaluated traits. Selection differentials for all traits were computed using a 20% selection intensity, which resulted in the selection of 33 of the 166 testcross hybrids. This selection intensity was chosen to align with the breeding programme’s advancement capacity of approximately 35 entries, including 30 experimental materials and five checks, while maintaining a manageable number of hybrids for subsequent multi-location and farmer-field evaluation. To assess the sensitivity of the MGIDI selection to selection intensity, additional analyses were performed using 10% and 30% selection intensities and compared with the 20% threshold. Consequently, accessions with lower MGIDI scores representing proximity to the ideal genotype were retained for selection. Following this, the proportional contribution of each factor to the MGIDI index was computed with Equation 8 to identify strengths and weaknesses of genotypes;

(8) $$\omega_{ij}=\frac{D_{ij}^{2}}{\sum_{j=1}^{f}D_{ij}^{2}}$$

ωij​ denotes the proportion of the MGIDI for the $i^{th}\;$ genotype that is explained by the correlated $j^{th}\;$factor, whereas ${\text{D}}_{\text{ij}}^{2}$​ represents the squared distance between the $i^{th}\;$ genotype and the ideotype with respect to the $j^{th}\;$factor.

Genotype plus genotype × environment (GGE) interaction analysis was conducted for grain yield on 33 selected testcross hybrids and the checks using the GGE biplot methodology described by Yan et al. (2000) and Yan and Kang (2003), as shown in Equation 9.

(9) $$Y_{ij}-\mu -\beta_{j}=\sum {\left(n=1\right)}^{N}\lambda_{n}\xi_{i}n\eta_{j}n+\epsilon_{i}j$$

where $Y_{ij}$ is the mean performance of genotype i in environment j, μ is the grand mean, $\beta_{j}$​ is the environmental effect, $\lambda_{n}$ is the singular value for principal component n, $\xi_{in}$ and $\eta_{jn}$​ are genotype and environment scores, and $\epsilon_{ij}$ is the residual.

Singular value decomposition (SVD) was used to partition the G + GE interaction, and the first two principal components (PC1 and PC2) were used for graphical interpretation.

## 3 Results

### 3.1 Analysis of variance

Genotypes varied significantly for ear rot (*p* ≤ 0.01), grain yield (*p* ≤ 0.0001), and other measured agronomic traits, except aflatoxin accumulation (**Table 2**). Similarly, there was significant variation among the environments for most of the traits including aflatoxin accumulation (*p* ≤ 0.05) and ear rot (*p* ≤ 0.01). In contrast, the effect of environment was not significant (*p* > 0.05) for grain yield. The interaction of environmental effects with genotype was significant (*p* ≤ 0.05) for days to anthesis, anthesis-silking interval, ear aspect, and plant height. It was, however, not significant (*p* > 0.05) for grain yield, aflatoxin accumulation, ear rot, days to silking, and ear height. Variance component estimates for genotypes were relatively higher compared to the estimate for G×E for most of the traits including grain yield, aflatoxin accumulation, days to anthesis, days to silking, plant height, and ear height. However, G×E variance exceeded the genotypic variance for ear rot and anthesis-silking interval, while both components contributed equally for ear aspect (**Table 3**). The proportional contribution of the genotypic variance to the total phenotypic variance ranged from 2.9% for aflatoxin accumulation to 35% for days to anthesis, while the contribution of G×E ranged from 0.4% for aflatoxin accumulation to 12.9% for anthesis-silking interval. The proportional contributions of genotypic variance for grain yield, ear rot, and aflatoxin accumulation were 15.2%, 6.7%, and 2.9%, respectively, while those of G×E were 7.6%, 11.1%, and 0.4%, respectively. Broad-sense heritability estimates for the traits varied from low to high. It was low for aflatoxin accumulation (H^2^ = 0.22), moderate for ear rot (H^2^ = 0.36), anthesis-silking interval, and ear aspect, and high for grain yield (H^2^ = 0.62), days to anthesis, days to silking, plant height, and ear height.

**Table 2:** Mean squares from the combined analysis of variance for grain yield, Aspergillus ear rot, aflatoxin accumulation, and agronomic traits of maize testcross across four contrasting environments.

Source of Variation	DF	GY	AFLT	AER	DTA	DTS	ASI	EA	PH	EH
Environment (Env)	3	171.18	403.47*	44.82**	2892.82***	3172.78***	205.01†	31.67†	165064***	17868**
Rep(Env)	4	36.10†	30.76†	1.77**	39.20†	37.62†	0.11	0.29	1731.91†	1067.08
Block(Rep×Env)	336	2.59	2.67	0.35	2.20**	2.61**	0.51	0.22*	186.29†	124.41†
Genotypes (G)	171	5.44†	2.30	0.54**	8.05†	8.05†	0.87***	0.34†	446.25†	293.31†
Env × G	511	2.30	2.22	0.38	1.94*	2.33	0.59**	0.21*	159.21**	85.85
Residual	346	2.20	2.31	0.35	1.64	2.02	0.44	0.17	123.49	74.06

*, **, ***, and † indicate significance at *p* ≤ 0.05, 0.01, 0.001, and 0.0001 respectively. DF: Degree of freedom; GY, Grain yield; AFLT, Aflatoxin accumulation (B1 + B2); AER, Aspergillus ear rot; EA, Ear aspect; ASI, Anthesis-silking interval; DTA, Days to anthesis; DTS, Days to silking; PH, Plant height; EH, Ear height.

**Table 3:** Estimates of variance components and genetic parameters for aflatoxin accumulation, Aspergillus ear rot, grain yield, and other agronomic traits of maize testcross hybrids across four contrasting environments.

Variance components	GY	AFLT	AER	DTA	DTS	ASI	EA	PH	EH
Genotype (σ²g)	0.51	0.08	0.03	1.25	1.17	0.06	0.03	51.80	36.98
G × E (σ²ge)	0.26	0.01	0.05	0.18	0.15	0.08	0.03	22.28	6.76
Residual (σ²e)	2.06	2.20	0.33	1.66	2.05	0.46	0.17	123.98	74.75
% σ²g	15.2	2.9	6.7	35.0	30.4	9.7	12.0	21.5	25.0
% σ²ge	7.6	0.4	11.1	5.0	3.9	12.9	12.0	9.3	4.6
H^2^	0.62	0.22	0.36	0.83	0.80	0.44	0.51	0.71	0.77

GY, Grain yield; AFLT, Aflatoxin accumulation (B1 + B2); AER, Aspergillus ear rot; EA, Ear aspect; ASI, Anthesis-silking interval; DTA, Days to anthesis; DTS, Days to silking; PH, Plant height; EH, Ear height; H^2^, Broad-sense heritability.

The mean performance of the testcross hybrids across the four environments for the measured traits is presented in **Table 4**. Grain yield of hybrids ranged from 5.89 t/ha in IK25 to 7.41 t/ha in IK24, that is at Ikenne in 2025 and 2024, respectively. The highest grain yield was recorded in IK24, although it was not statistically different from the other environments. The mean performance of the testcross hybrids for aflatoxin accumulation and ear rot varied considerably across the environments. Aflatoxin level was substantially high in IK24 (2, 320 ppb). In contrast, IB24 (229 ppb), IK25 (231 ppb), and IB25 (436 ppb) had relatively low and comparable aflatoxin levels. The highest ear rot severity was observed in IB25 (2.83), while IK24 had the lowest (2.0). Similar patterns were observed among the entries for the other measured traits.

**Table 4:** Mean performance of environments for grain yield, aflatoxin accumulation, Aspergillus ear rot, and some agronomic traits.

Environments	GY	AFLT	AER	EA	ASI	DTA	DTS	PH	EH
IB24	6.69	229.44a	2.47bc	2.94	3.16c	55.30bc	58.50c	247.00a	93.20b
IK24	7.41	2320.74b	2.00a	2.85	1.99b	50.40a	52.40a	207.00b	104.7a
IK25	5.89	231.75a	2.13ab	2.30	1.33a	53.40b	54.70b	190.00c	90.90b
IB25	7.34	436.03ab	2.83bc	2.91	1.71ab	57.60c	59.30c	215.00b	101.60ab
Statistics:									
SE	0.33	0.35	1.03	0.06	0.11	0.47	0.45	2.73	2.32
CV	28.1	29.7	31.4	20.4	53.0	5.9	6.1	11.9	14.5

Values with the same letter along the column are statistically the same. GY, Grain yield; AFLT, Aflatoxin accumulation (B1 + B2); AER, Aspergillus ear rot; EA, Ear aspect; ASI, Anthesis-silking interval; DTA, Days to anthesis; DTS, Days to silking; PH, Plant height; EH, Ear height; SE, Standard error; CV, Coefficient of variation.

### 3.2 Correlations between traits

The correlation between aflatoxin accumulation, ear rot, grain yield and agronomic parameters are shown in **Figure 1**. Aflatoxin accumulation had a significant positive correlation with ear rot (r = 0.24**). However, it was not significantly (*p >* 0.05) correlated with any of the agronomic traits, including grain yield (r = 0.08). Ear rot was significantly (*p* ≤ 0.05) and positively correlated with anthesis-silking interval and ear aspect, but significantly (*p* ≤ 0.05) and negatively associated with grain yield (r = -0.20*), ear height, and plant height. The correlations of ear rot with days to anthesis and days to silking were not significant. Similarly, grain yield had a positive and significant (*p* ≤ 0.01) correlation with ear height and plant height. In contrast, grain yield manifested significant (*p* ≤ 0.01) negative correlations with ear aspect, days to anthesis, and days to silking. The association between grain yield and anthesis-silking interval was not significant.

**Figure 1:**
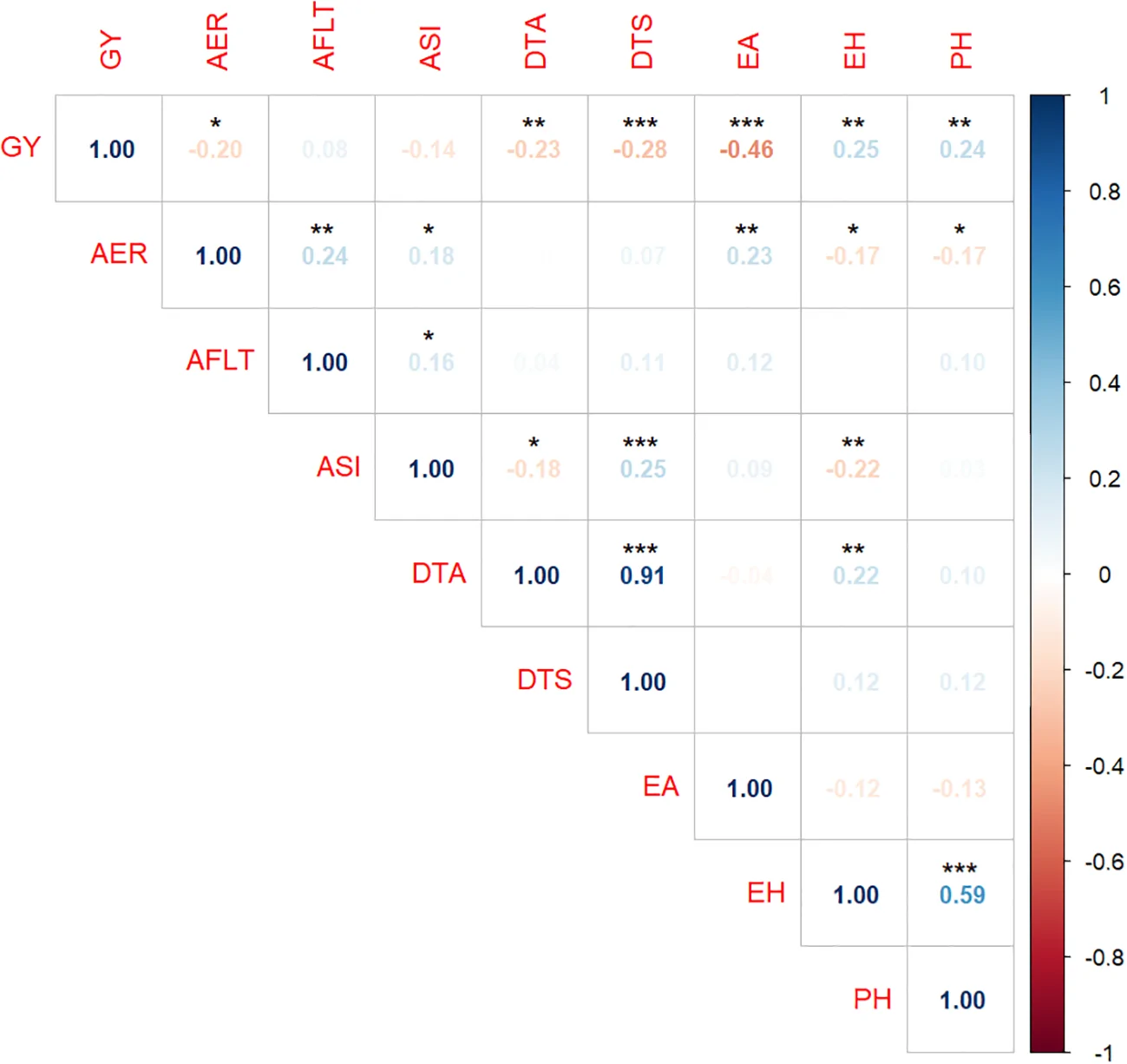
Pearson correlation among aflatoxin, aspergillus ear rot, grain yield, and some agronomic traits. *, **, and *** indicate significance at p ≤ 0.05, 0.01, and 0.001 respectively.

### 3.3 Multi-trait genotype-ideotype distance index selection and predicted selection gain

Variations among 166 testcross hybrids were assessed with nine traits. The MGIDI selected a total of 33 testcross hybrids, represented by the red dots, based on 20% selection intensity (**Figure 2**). The MGIDI analysis grouped the traits into three factors that collectively accounted for 68% of the total variation among the hybrids with an average uniqueness of 0.32 (**Table 5**). Factor 1 (FA1) was primarily associated with days to anthesis, days to silking, and ear height. While both days to anthesis and days to silking contributed positive loadings to FA1, ear height contributed negative loading. Factor 2 (FA2) comprised mainly of grain yield, ear aspect, ear height, and plant height, and contributed a strong negative loading. The third factor (FA3) was predominantly associated with disease-related traits, aflatoxin accumulation and ear rot, with positive contributions to the loading. Generally, all the traits showed encouraging predicted selection gain, aligning with the breeding objectives. Positive gain was observed for grain yield (0.58%), ear height (2.05%), and plant height (1.49%), while reductions were observed for aflatoxin accumulation (-0.04%), ear rot (-0.04%), days to anthesis (-0.33%), days to silking (-0.49%), ear aspect (-0.12%), and anthesis-silking interval (-0.11%) (**Table 5**).

**Figure 2:**
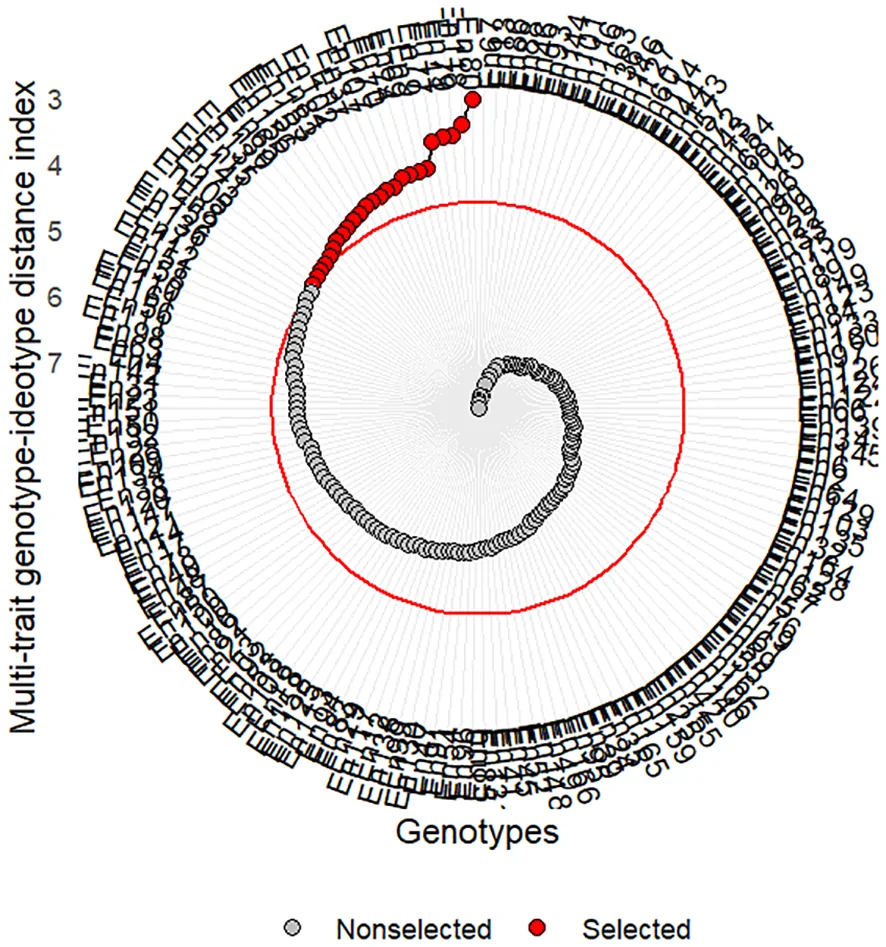
Maize testcross hybrids rankings showing selected accessions using the multi-trait genotype– ideotype index (MGIDI). The selected hybrids are represented by red dots, while the unchosen hybrids are shown as grey dots. The red circle indicates the cut-off point based on 20% selection intensity.

**Table 5:** Factorial loadings, communalities, uniqueness and selection gain based on multi-trait genotype-ideotype distance index of traits measured across four contrasting environments.

Traits	FA1	FA2	FA3	Communality	Uniqueness	SG%	Sense
GY	0.05	**-0.83**	0.03	0.69	0.31	0.58	Increase
AFLT	-0.01	0.04	**0.80**	0.64	0.36	-0.04	Decrease
AER	0.02	-0.25	**0.76**	0.64	0.36	-0.04	Decrease
ASI	-0.06	-0.39	0.28	0.23	0.77	-0.11	Decrease
DTA	**0.96**	0.12	-0.07	0.94	0.06	-0.33	Decrease
DTS	**0.97**	-0.00	0.02	0.94	0.06	-0.49	Decrease
EA	-0.06	**-0.71**	0.34	0.62	0.38	-0.12	Decrease
EH	**-0.55**	**-0.63**	-0.06	0.70	0.30	2.05	Increase
PH	-0.43	**-0.61**	-0.36	0.68	0.32	1.49	Increase
Average				0.68	0.32		

FA1, FA2, and FA3, factors 1, 2, and 3, respectively; SG, Selection gain, GY, Grain yield; AFLT, Aflatoxin accumulation (B1 + B2); AER, Aspergillus ear rot; EA, Ear aspect; ASI, Anthesis-silking interval; DTA, Days to anthesis; DTS, Days to silking; PH, Plant height; EH, Ear height. The values in bold indicate traits with high contribution to each factor loading.

### 3.4 BLUP estimate and MGIDI ranking

The best linear unbiased prediction (BLUP) estimates revealed considerable variation among the testcross hybrids for grain yield, aflatoxin accumulation, ear rot, and some agronomic traits. Aflatoxin levels ranged from 174.3 ppb (En74) to 269.5 ppb (En71), ear rot from 2.17 (En81) to 2.61 (En71), grain yield from 6.22 t/ha (En112) to 7.96 t/ha (En65) indicating large genetic variation (**Table 6**; **Supplementary Table 2**). In terms of grain yield, the three leading testcrosses were En65 (7.96 t/ha), En74 (7.88 t/ha), and En80 (7.83 t/ha). For aflatoxin accumulation, En74, En8, En9, En58, En37, En79, and En81 accumulated below 200 ppb. The differentiation among the testcrosses for ear rot score was not pronounced. Nevertheless, En81, En80, En37, En77, En7, and En107 had lower ear rot scores compared to others. When compared with the means of the genetic and commercial check hybrids, 29 testcross hybrids exceeded both check groups for grain yield (both means = 6.72 t/ha). Similarly, 14 testcross hybrids had lower ear rot scores than both check groups (both means = 2.39). For aflatoxin accumulation, 17 testcross hybrids accumulated less aflatoxin than the genetic check mean (216.54 ppb), while 14 accumulated less than the commercial check mean (213.84 ppb) (**Supplementary Table 2**).

**Table 6:** BLUP estimates and MGIDI-based ranking of top ten selected testcross hybrids and checks for grain yield, aflatoxin accumulation, and Aspergillus ear rot severity.

Testcrosses	AFLT	Rank based on aflatoxin	AER	GY	Rank based on yield	DTA	DTS	ASI	EA	PH	EH	MGIDI	Rank based on MGIDI
En80	211.57	11	2.20	7.83	3	55.21	58.02	2.99	2.68	241.29	96.63	3.01	1
En118	235.11	23	2.35	7.13	21	53.49	56.67	3.15	2.77	253.56	93.77	3.38	2
En79	191.95	6	2.31	6.72	30	53.75	56.62	2.99	2.64	235.92	88.77	3.55	3.5
En11	212.50	12	2.34	7.19	15	54.99	57.73	2.94	2.71	241.90	94.82	3.55	3.5
En77	204.18	8	2.27	7.28	12	55.33	58.24	3.05	2.71	251.07	96.87	3.59	5
En9	184.14	3	2.51	7.16	17.5	55.59	58.41	3.00	2.66	246.59	99.79	4.00	6.5
En69	257.78	31	2.28	6.83	29	53.69	56.66	3.05	2.83	236.37	89.57	4.00	6.5
En74	174.26	1	2.45	7.88	2	56.04	58.93	3.05	2.69	246.35	95.52	4.01	8
En70	261.51	32	2.34	7.27	13	54.92	58.32	3.27	2.67	244.93	93.41	4.03	9
En107	214.95	16	2.27	6.90	27	52.22	55.85	3.36	2.85	240.80	85.17	4.11	10
Genetic check mean	216.54		2.39	6.72		55.60	58.58	3.09	2.80	249.26	98.30		
Commercial check mean	213.84		2.39	6.72		56.19	59.5	3.27	2.99	250.66	101.29		

GY, Grain yield; AFLT, Aflatoxin accumulation (B1 + B2); AER, Aspergillus ear rot; EA, Ear aspect; ASI, Anthesis-silking interval; DTA, Days to anthesis; DTS, Days to silking; PH, Plant height; EH, Ear height; MGIDI, Multi-trait genotype-ideotype distance index.

The MGIDI integrated all the measured traits to assess the overall performance of the testcrosses, with lower values indicating superior overall desirability. The top five ranked testcrosses were En80, En118, En79, En11, and En77 with MGIDI values of 3.01, 3.38, 3.55, 3.55, and 3.59, respectively (**Table 6**). Importantly, top ranked testcross En80 combined high grain yield (7.83 t/ha) with moderate aflatoxin (211.6 ppb) and low ear rot (2.20), demonstrating a favourable balance between productivity and aflatoxin-related traits. Similarly, En9, En77, and En11 had relatively high grain yield with moderate aflatoxin levels. Notably, En74 ranked 2^nd^ for grain yield and had the lowest aflatoxin accumulation among the testcross hybrids but ranked 8^th^ based on MGIDI. Similarly, En58 and En81 ranked very highly for aflatoxin accumulation and had competitive grain yield, but ranked 31^st^ and 14^th^, respectively, by MGIDI. Likewise, En65 had the highest grain yield but ranked 18^th^ by MGIDI (**Supplementary Table 2**). Generally, the selected testcrosses showed competitive or superior performance relative to the checks. While the commercial check hybrid, Ife Hybrid 4 produced the highest grain yield (8.41 t/ha) among all the hybrids, the top selected testcrosses such as En80, En118, En79, En11, and En77 performed similar to the genetic check hybrid, En167 (T1×T2) and better than the other genetic and commercial check hybrids. Similar patterns were observed for both aflatoxin accumulation and ear rot scores (**Supplementary Table 2**).

The Spearman rank correlation analysis showed differences in the magnitude and direction of associations among MGIDI, grain yield, and aflatoxin accumulation (**Table 7**). There was a negative and non-significant association between MGIDI and grain yield (ρ = −0.10). Conversely, MGIDI had a positive and non-significant correlation with aflatoxin accumulation (ρ = 0.30). The association between grain yield and aflatoxin accumulation was positive and non-significant (ρ = 0.04).

**Table 7:** Spearman rank correlations among MGIDI, grain yield, and aflatoxin accumulation in 33 selected testcross hybrids.

Comparison	Spearman’s ρ	P-value
MGIDI vs Grain yield	-0.10	0.559
MGIDI vs Aflatoxin accumulation	0.30	0.09
Grain yield vs Aflatoxin accumulation	0.04	0.817

MGIDI, Multi-trait genotype-ideotype index.

A comprehensive overview of the strengths and weaknesses of each selected testcross hybrid based on the contribution of each factor to MGIDI is presented in **Figure 3**. The MGIDI contribution for each genotype was ranked from the most influential factor (located near the plot center) to the least influential factor (located farthest from the center). The black dotted lines indicate average performance in factor contribution. Higher factors moving toward the center indicate weaknesses, while lower values moving towards the plot periphery signify strengths. Testcross hybrids associated with FA1 such as En107, En69, En102, En152, En112, En79, and En118 showed strength in traits such as days to anthesis, days to silking, and ear height (**Figure 3**). For FA2, contributing testcross hybrids such as En65, En15, En73, En157, and En71 exhibited strengths for traits such as grain yield, ear aspect, ear height, and plant height. Similarly, En80, En58, En37, En81, En7, En74, En79, En11, En77, and En8 were associated with FA3 and demonstrated strong performance for traits such as aflatoxin accumulation and ear rot.

**Figure 3:**
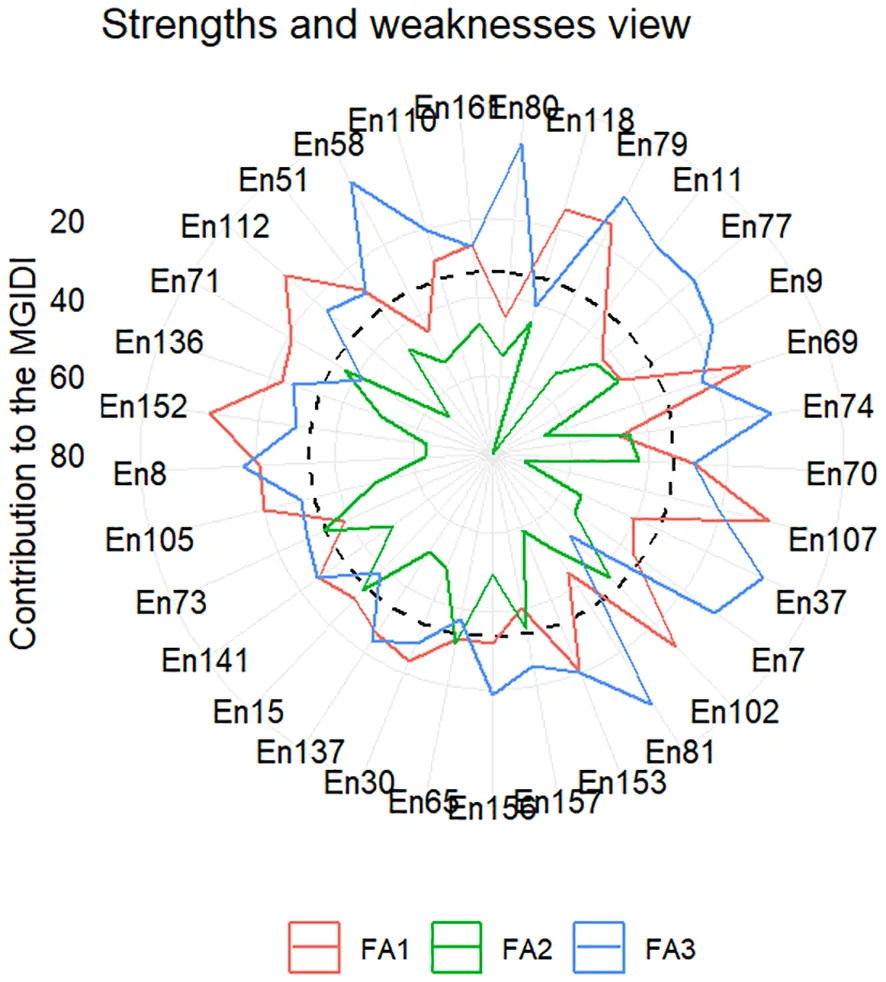
The strengths and weaknesses of the 33 selected testcross hybrids are depicted as the proportion of each factor on the computed multi-trait genotype-ideotype index (MGIDI). The black dashed line at the center shows theoretical value, assuming all factors contributed equally.

### 3.5 GGE biplot analysis

The GGE biplot showing the mean yield performance and stability of the selected testcross hybrids across four environments in Nigeria is shown in **Figure 4**. The first two principal components (PC1 and PC2) explained 76.4% of the total GGE variation among the selected hybrids, with PC1 and PC2 accounting for 51.21% and 25.17%, respectively. The biplot is composed of the average environments coordinates (AEC) abscissa and the AEC ordinate. The AEC abscissa, indicated by the arrow, ranked genotypes based on their mean yield performance. The genotypes located further in the direction of the arrow exhibited higher mean yield, while those further in the opposite direction of the arrow had low mean yield. Further, the projection of a genotype onto the AEC ordinate is a measure of stability with shorter projections indicating greater stability, while longer projections indicate stronger G×E, thus, reduced stability. The highest yielding genotype among the testcross hybrids was En65, followed closely by En74, En102, En80, En141, En157, En73, En71, and En136. These hybrids outperformed all checks, except En171 (Ife Hybrid 4) which recorded the highest overall mean yield. In contrast, En51 and En79 were the lowest yielding testcrosses, although their mean yields exceeded some of the commercial checks; En170 (Ife Hybrid 3) and En172 (SC645). In terms of stability, En80 and En65 exhibited the shortest projections from the AEC abscissa among the high-yielding testcrosses, indicating superior yield stability across the test environments. Similarly, En73, En74, En102, and En157 combined above average yield means with desirable stability. Although En15, En81, and En30 exhibited relatively high stability, they only had an average yield performance. Conversely, En79, En112, En9, En71, and En141 had long projections from the AEC abscissa, indicating the genotypes had very low stability across environments.

**Figure 4:**
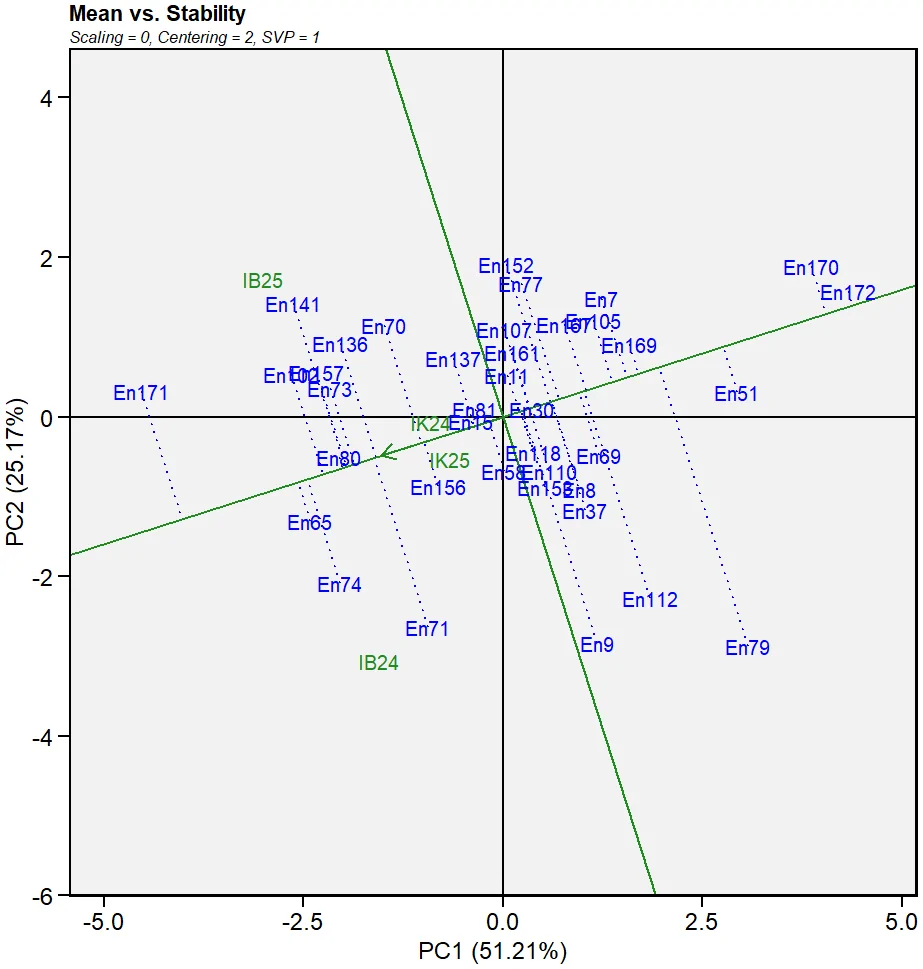
GGE biplot showing mean performance vs stability pattern of grain yield of 33 selected testcross hybrids and checks across four contrasting environments.

The “Which-won-where” view of the GGE biplot showed which genotype was winning in a particular environment. The polygon was formed by connecting the nine vertex genotypes; En171, En141, En152, En170, En172, En79, En9, En71, and En74 which were the farthest from the biplot origin, thereby enclosing all other genotypes within the polygon (**Figure 5**). Broken perpendicular lines were drawn from the biplot origin to the side of the polygon, which divided the biplot into nine sectors. Of all the nine sectors, only two contained test environments, leading to the identification of two mega-environments. The first mega-environment contained IB25, IK24, and IK25 which fell within the sector where En171 was the vertex genotype, indicating that En171 was the winning genotype in these environments. The second mega-environment had only IB24, which was on the boundary of sectors where En74 and En71 were the vertex genotypes, suggesting comparable performance of both genotypes in the environment. Although nine genotypes formed the vertices of the polygon, only three (En171, En74, and En71) were associated with sectors containing test environments.

**Figure 5:**
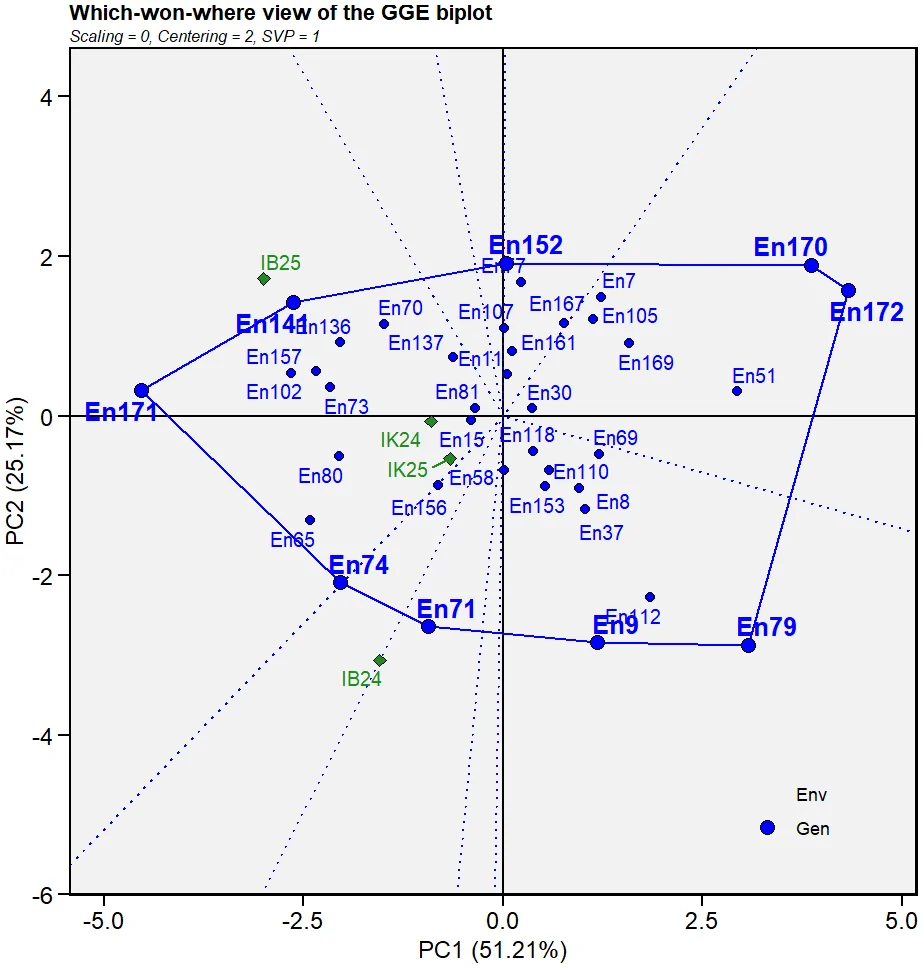
The “Which-won-where” biplot of PC1 and PC2 showing which of the 33 selected testcross hybrids and checks performed best in which test environments.

## 4 Discussion

The significant differences among the hybrids that were observed for grain yield, ear rot, and several agronomic traits indicate the presence of exploitable genetic variability among the testcrosses. This suggests that genetic improvement through selection of hybrids with the desired traits would be feasible (Oppong et al., 2022). In contrast, the absence of significant genotypic differences for aflatoxin accumulation indicates challenges in developing resistant hybrids. This partly reflects the quantitative and complex nature of aflatoxin accumulation, which is influenced by genetic and environmental factors (Brown et al., 2013; Warburton and Williams, 2014). In addition, the limited genetic differentiation observed for aflatoxin accumulation may be due to the common genetic background of the parental inbred lines, which were all derived through marker-assisted introgression of aflatoxin-resistant QTLs from the same donor source. The shared resistance alleles among the parental lines may have reduced the genetic contrast for aflatoxin accumulation among the resulting testcross hybrids, thereby limiting the detection of significant genotypic differences (Collard and Mackill, 2007; Xu and Crouch, 2008). The significant environmental effects on ear rot, aflatoxin accumulation and several other agronomic traits further demonstrated the strong influence of environmental conditions on the expression of these traits. This was notable in IK24 where the testcrosses accumulated substantially higher aflatoxin levels compared to other environments. These results corroborate the earlier reports that environmental conditions are major determinants of aflatoxin accumulation in maize (Okoth et al., 2017; Nji et al., 2024; Bereziartua et al., 2025). Although environmental effects were significant for ear rot and aflatoxin accumulation, G×E was not significant for either trait in the present study. This indicates that environmental conditions strongly influenced the expression of these traits, but the relative responses of the testcrosses did not differ sufficiently among the evaluated environments to produce a statistically detectable interaction. Thus, selection for these traits can be based on overall genotype performance across the evaluated environments. This is consistent with previous findings (Oppong et al., 2022, 2023; Mesterházy et al., 2024). In contrast to earlier reports that attributed greater proportion of variation in aflatoxin accumulation to G×E (Okoth et al., 2017; Oppong et al., 2023), genotypic variance for aflatoxin accumulation was more than G×E in this study, although both components were low in magnitude. The observed genotypic variance may partly reflect the influence of favourable resistance alleles introgressed into the parental inbred lines, which were previously confirmed through KASP genotyping to carry aflatoxin-resistant QTLs (Diekade et al., 2026). However, the transmission and expression of these loci were not directly validated in the testcross hybrids. Therefore, the observed variation in aflatoxin accumulation should be interpreted as phenotypic evidence from hybrid field evaluation rather than direct evidence of QTL effects. Previous studies have shown that QTL introgressed into parental lines can remain functional and contribute to trait expression in both advanced lines and derived hybrids (Kumar et al., 2018; Meseka et al., 2018; Kumar et al., 2023; Moretzsohn et al., 2023). However, the specific contributions of the targeted QTLs to the performance of the evaluated hybrids requires further genotypic investigation.

The relatively low to moderate broad-sense heritability estimates for aflatoxin accumulation and ear rot further indicate strong environmental influence on the expression of the traits. This is consistent with the previous reports that both traits exhibit low to moderate heritability (Warburton and Williams, 2014; Okoth et al., 2017). These findings reinforce the importance of multi-environment evaluation and the use of appropriate selection approaches when improving resistance-related traits. However, the relatively high heritability observed for grain yield provides greater opportunity for effective selection for productivity.

The positive correlation observed between aflatoxin accumulation and ear rot suggests that increased fungal colonization may increase the likelihood of higher aflatoxin accumulation. However, the relatively weak association implies that visual disease symptoms may not be reliably used to predict aflatoxin levels in maize genotypes, highlighting the need for direct screening of hybrids for low aflatoxin accumulation. Similar observations of weak correlation between these traits were reported in previous studies (Okoth et al., 2017; Barošević et al., 2022; Mboup et al., 2023, 2024). Moreover, it has been reported that the resistance to aflatoxin accumulation and resistance to ear rot are partially under independent genetic control (Widstrom, 1996; Warburton and Williams, 2014). The lack of significant association between aflatoxin accumulation and grain yield suggests that simultaneous improvement of both traits without necessarily compromising yield potential could be possible. This agrees with previous reports that high grain yield and low aflatoxin accumulation are not mutually exclusive breeding objectives (Warburton and Williams, 2014; Mutiga et al., 2017). In contrast, the significant negative correlation between ear rot and grain yield indicates that greater disease severity on ear or kernel may be associated with reduced productivity, reinforcing the importance of controlling ear rot alongside grain yield improvement. In a recent multi-locational trial, Abdallah et al. (2026) reported that ear rot damages kernels and disrupts physiological processes, ultimately reducing grain quality and productivity.

The MGIDI analysis enabled selection based on the simultaneous consideration of productivity, resistance-related, and agronomic traits. The selection of 33 testcross hybrids at 20% selection intensity therefore provided a subset of materials with a more balanced combination of the evaluated traits. Previous studies have reported the effectiveness of MGIDI in identifying maize genotypes with superior performance across multiple desirable traits (Singamsetti et al., 2023; Azrai et al., 2025; Channapur et al., 2026; Kuku et al., 2026). Among the top selected testcrosses, En80 combined competitive grain yield with relatively low aflatoxin accumulation, and had the lowest MGIDI value, indicating its proximity to the ideotype. Similarly, En74, En102, and En157 combined high grain yield with favourable performance for aflatoxin accumulation and ranked relatively highly among the MGIDI-selected testcrosses. These testcrosses therefore represent promising candidates for advancement and further evaluation. However, the MGIDI ranking also illustrates the trade-offs associated with multi-trait selection. For example, En65 exhibited the highest grain yield but ranked 18^th^ by MGIDI. Similarly, En74 had the lowest aflatoxin accumulation but ranked eighth. This indicates that outstanding performance for an individual trait does not necessarily translate into the best overall MGIDI ranking when multiple breeding objectives are considered simultaneously.

The predicted selection gains further illustrate the expected response from MGIDI-based selection. Positive gains for grain yield and negative gains for aflatoxin accumulation, ear rot, and anthesis-silking interval were in the desired directions according to the predefined breeding objectives. However, these values represent predicted responses under the fitted model and should not be interpreted as realized genetic gains. An important limitation of MGIDI is that the resulting ranking depends on the traits included in the index, their predefined desired directions, and the selected intensity. Genotypes with exceptional performance for one trait may therefore receive lower overall rankings if they perform less favourably for other traits. MGIDI should consequently be regarded as a multi-trait decision-support tool for balancing predefined breeding objectives rather than as an absolute measure of hybrid superiority.

Following the identification of the 33 testcross hybrids with desired multi-trait profiles by MGIDI, GGE biplot analysis was used to further characterize their grain yield performance, stability, and adaptation across environments. The GGE analysis therefore provided additional information on grain yield performance that could not be obtained from MGIDI alone. The identification of two distinct mega-environments within the evaluated subset indicates differential adaptation among the selected testcross hybrids and suggests that genotype recommendations may need to be targeted to specific production environments. The association of En171 with environments IB25, IK24, and IK25, and the positioning of environment IB24 near the boundary between En74, and En71 further emphasizes the importance of considering specific adaptation when selecting superior hybrids.

The GGE biplot identified En65 as the top performer for grain yield, although Ife Hybrid 4 (En171), a commercial hybrid, remained the highest-yielding hybrid. This indicates that there is still considerable scope to improve the testcross hybrids for grain yield under the evaluated conditions. Nevertheless, the relatively shorter projections from the AEC abscissa exhibited by some selected testcross hybrids, including En80 and En65, indicate greater stability than Ife Hybrid 4. Thus, some of the selected testcrosses combined competitive grain yield with greater stability, highlighting the importance of considering both performance and stability rather than yield alone when selecting hybrids for broader adaptation. Similar emphasis on selection of high-yielding and stable maize hybrids using GGE biplot has been reported (Gonçalves et al., 2025). According to Liu et al. (2022) and Ma et al. (2024), an important objective of modern maize hybrid development is not only to identify genotypes with high grain yield, but also those that combine competitive grain yield with stable performance across diverse production environments. The contrasting performance of En65 between the two analytical approaches further illustrates their complementary roles. Its superior grain yield and favourable stability were revealed through GGE biplot, whereas its lower MGIDI ranking reflected its less balanced performance across the broader set of breeding objectives. Similar differences between MGIDI- and GGE-based ranking were observed by Susilawati et al. (2026) who reported that the genotype ranked best by GGE biplot was not selected by MGIDI approach. The superior performance of several selected testcross hybrids relative to the genetic and commercial check groups further support their potential for continued evaluation. Given that the GGE analysis was conducted only on the MGIDI-selected testcrosses, the observed stability and adaptability patterns should be interpreted within this pre-selected group and not generalized to the entire set of 166 testcrosses. Overall, the sequential use of MGIDI and GGE provided complementary information, with MGIDI identifying testcrosses with balanced multi-trait performance and GGE subsequently assessing yield performance, stability, and adaptation among the selected genotypes. This combination is particularly useful where breeding decisions require simultaneous consideration of multiple traits and environmental performance.

## 5 Conclusion

The current study demonstrated that ear rot and aflatoxin accumulation in maize were strongly influenced by environmental conditions, highlighting the importance of multi-environment evaluation for effective selection. The relatively low to moderate heritability estimates observed for aflatoxin accumulation and ear rot further confirm the complexity of improving these traits and emphasize the need for robust analytical approaches to enhance selection efficiency. Although ear rot showed a positive significant association with aflatoxin accumulation, the low magnitude of the correlation suggests that visual disease severity alone may not be a reliable predictor of grain aflatoxin content, reinforcing the importance of direct phenotypic screening.

The sequential use of MGIDI and GGE provided a complementary framework for identifying superior testcrosses by simultaneously considering productivity, stability, and resistance-related traits. While MGIDI enabled selection of testcrosses with more balanced performance across the predefined breeding objectives, GGE biplot further characterized grain yield performance, stability, and adaptation among the selected testcross hybrids. The integrated approach identified En80, En74, En102 En157, and En65 as promising testcross hybrids that combined competitive grain yield with relatively low aflatoxin accumulation and favourable stability, while differences in MGIDI ranking reflected the broader range of traits considered in multi-trait selection. These hybrids represent promising genetic resources for advancement to multi-location evaluation. Given that GGE analysis was conducted only on the MGIDI-selected testcrosses and checks, the observed stability and adaptation patterns should be interpreted within this pre-selected group. Further evaluation across broader environments will be necessary to determine the consistency of their performance and potential for advancement in maize hybrid development. The phenotypic performance observed in this study also supports the continued evaluation of QTL-introgressed parental materials for developing maize hybrids with reduced aflatoxin accumulation and improved productivity.

## Data Availability

The original contributions presented in the study are included in the article/**Supplementary Material**. Further inquiries can be directed to the corresponding authors.
