# Supplementary material for: Multi-trait selection of tropical maize hybrids for combined productivity, agronomic stability, and aflatoxin resistance
Source: Front Plant Sci. 2026 Sep 30;17:1964715. doi: 10.3389/fpls.2026.1964715 (PMC13662164; doi:10.3389/fpls.2026.1964715)
Supplement: Supplementary file 1 [file Table1.docx]

Supplementary Table 1. Testcross hybrids of yellow-kernel inbred lines enriched with aflatoxin-resistant QTLs and checks evaluated across four contrasting environments in this study

| **Entries** | **Pedigree** |
| --- | --- |
| Testcrosses |  |
| En1 | (IITATZI1394 /Mp715/(9450/CM116/9450)-3-3-1-2-1*7/9450/KI21-1-5-3-2-2/DE3/9450/KI21-1-5-3-2-2-30B-19-1) -1-2-1/SYN-Y-STR-34-1-1-1-1-2-1BB/KU1409xMO17LPAxKU1409-27-3-1-1-15*9 |
| En2 | (IITATZI1394 /Mp715/(9450/CM116/9450)-3-3-1-2-1*7/9450/KI21-1-5-3-2-2/DE3/9450/KI21-1-5-3-2-2-30B-19-1) -4-3-1/SYN-Y-STR-34-1-1-1-1-2-1BB/KU1409xMO17LPAxKU1409-27-3-1-1-15*9 |
| En3 | (IITATZI1394 /Mp715/(9450/CM116/9450)-3-3-1-2-1*7/9450/KI21-1-5-3-2-2/DE3/9450/KI21-1-5-3-2-2-30B-19-1) -6-2-1-1-2-1/SYN-Y-STR-34-1-1-1-1-2-1BB/KU1409xMO17LPAxKU1409-27-3-1-1-15*9 |
| En4 | (IITATZI1394 /Mp715/(9450/CM116/9450)-3-3-1-2-1*7/9450/KI21-1-5-3-2-2/DE3/9450/KI21-1-5-3-2-2-30B-19-1) -6-2-1-1-2-2/SYN-Y-STR-34-1-1-1-1-2-1BB/KU1409xMO17LPAxKU1409-27-3-1-1-15*9 |
| En5 | (IITATZI1394 /Mp715/(9450/CM116/9450)-3-3-1-2-1*7/9450/KI21-1-5-3-2-2/DE3/9450/KI21-1-5-3-2-2-30B-19-1) -6-4-1/SYN-Y-STR-34-1-1-1-1-2-1BB/KU1409xMO17LPAxKU1409-27-3-1-1-15*9 |
| En6 | (IITATZI1394 /Mp715/(9450/CM116/9450)-3-3-1-2-1*7/9450/KI21-1-5-3-2-2/DE3/9450/KI21-1-5-3-2-2-30B-19-1) -6-4-2-1-1/SYN-Y-STR-34-1-1-1-1-2-1BB/KU1409xMO17LPAxKU1409-27-3-1-1-15*9 |
| En7 | (IITATZI1394 /Mp715/(9450/CM116/9450)-3-3-1-2-1*7/9450/KI21-1-5-3-2-2/DE3/9450/KI21-1-5-3-2-2-30B-19-1) -5-3-2/SYN-Y-STR-34-1-1-1-1-2-1BB/KU1409xMO17LPAxKU1409-27-3-1-1-15*9 |
| En8 | (IITATZI1394 /Mp715/(9450/CM116/9450)-3-3-1-2-1*7/9450/KI21-1-5-3-2-2/DE3/9450/KI21-1-5-3-2-2-30B-19-1) -1-1-1/SYN-Y-STR-34-1-1-1-1-2-1BB/KU1409xMO17LPAxKU1409-27-3-1-1-15*9 |
| En9 | (IITATZI1394 /Mp715/(9450/CM116/9450)-3-3-1-2-1*7/9450/KI21-1-5-3-2-2/DE3/9450/KI21-1-5-3-2-2-30B-19-1) -2-1-1-1/SYN-Y-STR-34-1-1-1-1-2-1BB/KU1409xMO17LPAxKU1409-27-3-1-1-15*9 |
| En10 | (IITATZI1394 /Mp715/(9450/CM116/9450)-3-3-1-2-1*7/9450/KI21-1-5-3-2-2/DE3/9450/KI21-1-5-3-2-2-30B-19-1) -2-1-2-1-1/SYN-Y-STR-34-1-1-1-1-2-1BB/KU1409xMO17LPAxKU1409-27-3-1-1-15*9 |
| En11 | (IITATZI1394 /Mp715/(9450/CM116/9450)-3-3-1-2-1*7/9450/KI21-1-5-3-2-2/DE3/9450/KI21-1-5-3-2-2-30B-19-1) -2-1-2-1-2/SYN-Y-STR-34-1-1-1-1-2-1BB/KU1409xMO17LPAxKU1409-27-3-1-1-15*9 |
| En12 | (IITATZI1394 /Mp715/(9450/CM116/9450)-3-3-1-2-1*7/9450/KI21-1-5-3-2-2/DE3/9450/KI21-1-5-3-2-2-30B-19-1) -2-1-2-1-3/SYN-Y-STR-34-1-1-1-1-2-1BB/KU1409xMO17LPAxKU1409-27-3-1-1-15*9 |
| En13 | (IITATZI1394 /Mp715/(9450/CM116/9450)-3-3-1-2-1*7/9450/KI21-1-5-3-2-2/DE3/9450/KI21-1-5-3-2-2-30B-19-1) -1-1-2-1-1/SYN-Y-STR-34-1-1-1-1-2-1BB/KU1409xMO17LPAxKU1409-27-3-1-1-15*9 |
| En14 | (IITATZI1394 /Mp715/(9450/CM116/9450)-3-3-1-2-1*7/9450/KI21-1-5-3-2-2/DE3/9450/KI21-1-5-3-2-2-30B-19-1) -1-1-2-1-2/SYN-Y-STR-34-1-1-1-1-2-1BB/KU1409xMO17LPAxKU1409-27-3-1-1-15*9 |
| En15 | (IITATZI1394 /Mp715/(9450/CM116/9450)-3-3-1-2-1*7/9450/KI21-1-5-3-2-2/DE3/9450/KI21-1-5-3-2-2-30B-19-1) -1-1-2-2/SYN-Y-STR-34-1-1-1-1-2-1BB/KU1409xMO17LPAxKU1409-27-3-1-1-15*9 |
| En16 | (IITATZI1394 /Mp715/(9450/CM116/9450)-3-3-1-2-1*7/9450/KI21-1-5-3-2-2/DE3/9450/KI21-1-5-3-2-2-30B-19-1) -1-1-2-3/SYN-Y-STR-34-1-1-1-1-2-1BB/KU1409xMO17LPAxKU1409-27-3-1-1-15*9 |
| En17 | (IITATZI1394 /Mp715/(9450/CM116/9450)-3-3-1-2-1*7/9450/KI21-1-5-3-2-2/DE3/9450/KI21-1-5-3-2-2-30B-19-1) -1-1-1-3/SYN-Y-STR-34-1-1-1-1-2-1BB/KU1409xMO17LPAxKU1409-27-3-1-1-15*9 |
| En18 | (IITATZI1394 /Mp715/(9450/CM116/9450)-3-3-1-2-1*7/9450/KI21-1-5-3-2-2/DE3/9450/KI21-1-5-3-2-2-30B-19-1) -1-1-1-4/SYN-Y-STR-34-1-1-1-1-2-1BB/KU1409xMO17LPAxKU1409-27-3-1-1-15*9 |
| En19 | (IITATZI1394 /Mp715/(9450/CM116/9450)-3-3-1-2-1*7/9450/KI21-1-5-3-2-2/DE3/9450/KI21-1-5-3-2-2-30B-19-1) -1-1-2-1/SYN-Y-STR-34-1-1-1-1-2-1BB/KU1409xMO17LPAxKU1409-27-3-1-1-15*9 |
| En20 | (IITATZI1394 /Mp715/(9450/CM116/9450)-3-3-1-2-1*7/9450/KI21-1-5-3-2-2/DE3/9450/KI21-1-5-3-2-2-30B-19-1) -2-2-1-1/SYN-Y-STR-34-1-1-1-1-2-1BB/KU1409xMO17LPAxKU1409-27-3-1-1-15*9 |
| En21 | (IITATZI1394 /Mp715/(9450/CM116/9450)-3-3-1-2-1*7/9450/KI21-1-5-3-2-2/DE3/9450/KI21-1-5-3-2-2-30B-19-1) -5-1-1-2/SYN-Y-STR-34-1-1-1-1-2-1BB/KU1409xMO17LPAxKU1409-27-3-1-1-15*9 |
| En22 | (IITATZI1394 /Mp715/(9450/CM116/9450)-3-3-1-2-1*7/9450/KI21-1-5-3-2-2/DE3/9450/KI21-1-5-3-2-2-30B-19-1) -6-5-1-1/SYN-Y-STR-34-1-1-1-1-2-1BB/KU1409xMO17LPAxKU1409-27-3-1-1-15*9 |
| En23 | (IITATZI1394 /Mp715/(9450/CM116/9450)-3-3-1-2-1*7/9450/KI21-1-5-3-2-2/DE3/9450/KI21-1-5-3-2-2-30B-19-1) -1-1-1-2/SYN-Y-STR-34-1-1-1-1-2-1BB/KU1409xMO17LPAxKU1409-27-3-1-1-15*9 |
| En24 | (IITATZI1394 /Mp715/(9450/CM116/9450)-3-3-1-2-1*7/9450/KI21-1-5-3-2-2/DE3/9450/KI21-1-5-3-2-2-30B-19-1) -7-1-1-2/SYN-Y-STR-34-1-1-1-1-2-1BB/KU1409xMO17LPAxKU1409-27-3-1-1-15*9 |
| En25 | (IITATZI1394 /Mp715/(9450/CM116/9450)-3-3-1-2-1*7/9450/KI21-1-5-3-2-2/DE3/9450/KI21-1-5-3-2-2-30B-19-1) -9-3-1-1/SYN-Y-STR-34-1-1-1-1-2-1BB/KU1409xMO17LPAxKU1409-27-3-1-1-15*9 |
| En26 | (IITATZI1394 /Mp715/(9450/CM116/9450)-3-3-1-2-1*7/9450/KI21-1-5-3-2-2/DE3/9450/KI21-1-5-3-2-2-30B-19-1) -10-3-2-1/SYN-Y-STR-34-1-1-1-1-2-1BB/KU1409xMO17LPAxKU1409-27-3-1-1-15*9 |
| En27 | (IITATZI1394 /Mp715/(9450/CM116/9450)-3-3-1-2-1*7/9450/KI21-1-5-3-2-2/DE3/9450/KI21-1-5-3-2-2-30B-19-1) -10-3-3-2/SYN-Y-STR-34-1-1-1-1-2-1BB/KU1409xMO17LPAxKU1409-27-3-1-1-15*9 |
| En28 | (IITATZI1394 /Mp715/(9450/CM116/9450)-3-3-1-2-1*7/9450/KI21-1-5-3-2-2/DE3/9450/KI21-1-5-3-2-2-30B-19-1) -1-1-3-1/SYN-Y-STR-34-1-1-1-1-2-1BB/KU1409xMO17LPAxKU1409-27-3-1-1-15*9 |
| En29 | (IITATZI1394 /Mp715/(9450/CM116/9450)-3-3-1-2-1*7/9450/KI21-1-5-3-2-2/DE3/9450/KI21-1-5-3-2-2-30B-19-1) -2-2-1-1/SYN-Y-STR-34-1-1-1-1-2-1BB/KU1409xMO17LPAxKU1409-27-3-1-1-15*9 |
| En30 | (IITATZI1394 /Mp715/(9450/CM116/9450)-3-3-1-2-1*7/9450/KI21-1-5-3-2-2/DE3/9450/KI21-1-5-3-2-2-30B-19-1) -6-1-2-1/SYN-Y-STR-34-1-1-1-1-2-1BB/KU1409xMO17LPAxKU1409-27-3-1-1-15*9 |
| En31 | (IITATZI1394 /Mp715/(9450/CM116/9450)-3-3-1-2-1*7/9450/KI21-1-5-3-2-2/DE3/9450/KI21-1-5-3-2-2-30B-19-1) -6-1-2-2/SYN-Y-STR-34-1-1-1-1-2-1BB/KU1409xMO17LPAxKU1409-27-3-1-1-15*9 |
| En32 | (IITATZI1394 /Mp715/(9450/CM116/9450)-3-3-1-2-1*7/9450/KI21-1-5-3-2-2/DE3/9450/KI21-1-5-3-2-2-30B-19-1) -3-1-1-1/SYN-Y-STR-34-1-1-1-1-2-1BB/KU1409xMO17LPAxKU1409-27-3-1-1-15*9 |
| En33 | (IITATZI1394 /Mp715/(9450/CM116/9450)-3-3-1-2-1*7/9450/KI21-1-5-3-2-2/DE3/9450/KI21-1-5-3-2-2-30B-19-1) -3-1-1-2/SYN-Y-STR-34-1-1-1-1-2-1BB/KU1409xMO17LPAxKU1409-27-3-1-1-15*9 |
| En34 | (IITATZI1394 /Mp715/(9450/CM116/9450)-3-3-1-2-1*7/9450/KI21-1-5-3-2-2/DE3/9450/KI21-1-5-3-2-2-30B-19-1) -2-4-1-2/SYN-Y-STR-34-1-1-1-1-2-1BB/KU1409xMO17LPAxKU1409-27-3-1-1-15*9 |
| En35 | (IITATZI1394 /Mp715/(9450/CM116/9450)-3-3-1-2-1*7/9450/KI21-1-5-3-2-2/DE3/9450/KI21-1-5-3-2-2-30B-19-1) -2-1-2-1/SYN-Y-STR-34-1-1-1-1-2-1BB/KU1409xMO17LPAxKU1409-27-3-1-1-15*9 |
| En36 | (IITATZI1394 /Mp715/(9450/CM116/9450)-3-3-1-2-1*7/9450/KI21-1-5-3-2-2/DE3/9450/KI21-1-5-3-2-2-30B-19-1) -2-2-1-1/SYN-Y-STR-34-1-1-1-1-2-1BB/KU1409xMO17LPAxKU1409-27-3-1-1-15*9 |
| En37 | (IITATZI1394 /Mp715/(9450/CM116/9450)-3-3-1-2-1*7/9450/KI21-1-5-3-2-2/DE3/9450/KI21-1-5-3-2-2-30B-19-1) -2-2-1-2-1/SYN-Y-STR-34-1-1-1-1-2-1BB/KU1409xMO17LPAxKU1409-27-3-1-1-15*9 |
| En38 | (IITATZI1394 /Mp715/(9450/CM116/9450)-3-3-1-2-1*7/9450/KI21-1-5-3-2-2/DE3/9450/KI21-1-5-3-2-2-30B-19-1) -2-2-1-2-2/SYN-Y-STR-34-1-1-1-1-2-1BB/KU1409xMO17LPAxKU1409-27-3-1-1-15*9 |
| En39 | (IITATZI1394 /Mp715/(9450/CM116/9450)-3-3-1-2-1*7/9450/KI21-1-5-3-2-2/DE3/9450/KI21-1-5-3-2-2-30B-19-1) -2-2-1-1/SYN-Y-STR-34-1-1-1-1-2-1BB/KU1409xMO17LPAxKU1409-27-3-1-1-15*9 |
| En40 | (IITATZI1394 /Mp715/(9450/CM116/9450)-3-3-1-2-1*7/9450/KI21-1-5-3-2-2/DE3/9450/KI21-1-5-3-2-2-30B-19-1) -4-2-1-1/SYN-Y-STR-34-1-1-1-1-2-1BB/KU1409xMO17LPAxKU1409-27-3-1-1-15*9 |
| En41 | (IITATZI1394 /Mp715/(9450/CM116/9450)-3-3-1-2-1*7/9450/KI21-1-5-3-2-2/DE3/9450/KI21-1-5-3-2-2-30B-19-1) -1-2-1-1-1/SYN-Y-STR-34-1-1-1-1-2-1BB/KU1409xMO17LPAxKU1409-27-3-1-1-15*9 |
| En42 | (IITATZI1394 /Mp715/(9450/CM116/9450)-3-3-1-2-1*7/9450/KI21-1-5-3-2-2/DE3/9450/KI21-1-5-3-2-2-30B-19-1) -1-2-1-1-2/SYN-Y-STR-34-1-1-1-1-2-1BB/KU1409xMO17LPAxKU1409-27-3-1-1-15*9 |
| En43 | (IITATZI1394 /Mp715/(9450/CM116/9450)-3-3-1-2-1*7/9450/KI21-1-5-3-2-2/DE3/9450/KI21-1-5-3-2-2-30B-19-1) -1-1-1-1-1/SYN-Y-STR-34-1-1-1-1-2-1BB/KU1409xMO17LPAxKU1409-27-3-1-1-15*9 |
| En44 | (IITATZI1394 /Mp715/(9450/CM116/9450)-3-3-1-2-1*7/9450/KI21-1-5-3-2-2/DE3/9450/KI21-1-5-3-2-2-30B-19-1) -1-1-1-1-2/SYN-Y-STR-34-1-1-1-1-2-1BB/KU1409xMO17LPAxKU1409-27-3-1-1-15*9 |
| En45 | (IITATZI1394 /Mp715/(9450/CM116/9450)-3-3-1-2-1*7/9450/KI21-1-5-3-2-2/DE3/9450/KI21-1-5-3-2-2-30B-19-1) -2-2-1-2-1/SYN-Y-STR-34-1-1-1-1-2-1BB/KU1409xMO17LPAxKU1409-27-3-1-1-15*9 |
| En46 | (IITATZI1394 /Mp715/(9450/CM116/9450)-3-3-1-2-1*7/9450/KI21-1-5-3-2-2/DE3/9450/KI21-1-5-3-2-2-30B-19-1) -2-2-1-2-2/SYN-Y-STR-34-1-1-1-1-2-1BB/KU1409xMO17LPAxKU1409-27-3-1-1-15*9 |
| En47 | (IITATZI1394 /Mp715/(9450/CM116/9450)-3-3-1-2-1*7/9450/KI21-1-5-3-2-2/DE3/9450/KI21-1-5-3-2-2-30B-19-1) -2-1-2-1/SYN-Y-STR-34-1-1-1-1-2-1BB/KU1409xMO17LPAxKU1409-27-3-1-1-15*9 |
| En48 | (IITATZI1394 /Mp715/(9450/CM116/9450)-3-3-1-2-1*7/9450/KI21-1-5-3-2-2/DE3/9450/KI21-1-5-3-2-2-30B-19-1) -2-1-2-2/SYN-Y-STR-34-1-1-1-1-2-1BB/KU1409xMO17LPAxKU1409-27-3-1-1-15*9 |
| En49 | (IITATZI1394 /Mp715/(9450/CM116/9450)-3-3-1-2-1*7/9450/KI21-1-5-3-2-2/DE3/9450/KI21-1-5-3-2-2-30B-19-1) -2-1-2-3-1/SYN-Y-STR-34-1-1-1-1-2-1BB/KU1409xMO17LPAxKU1409-27-3-1-1-15*9 |
| En50 | (IITATZI1394 /Mp715/(9450/CM116/9450)-3-3-1-2-1*7/9450/KI21-1-5-3-2-2/DE3/9450/KI21-1-5-3-2-2-30B-19-1) -2-1-2-3-2/SYN-Y-STR-34-1-1-1-1-2-1BB/KU1409xMO17LPAxKU1409-27-3-1-1-15*9 |
| En51 | (IITATZI1394 /Mp715/(9450/CM116/9450)-3-3-1-2-1*7/9450/KI21-1-5-3-2-2/DE3/9450/KI21-1-5-3-2-2-30B-19-1) -4-1-1-3/SYN-Y-STR-34-1-1-1-1-2-1BB/KU1409xMO17LPAxKU1409-27-3-1-1-15*9 |
| En52 | (IITATZI1394 /Mp715/(9450/CM116/9450)-3-3-1-2-1*7/9450/KI21-1-5-3-2-2/DE3/9450/KI21-1-5-3-2-2-30B-19-1) -5-2-1-2/SYN-Y-STR-34-1-1-1-1-2-1BB/KU1409xMO17LPAxKU1409-27-3-1-1-15*9 |
| En53 | (IITATZI1394 /Mp715/(9450/CM116/9450)-3-3-1-2-1*7/9450/KI21-1-5-3-2-2/DE3/9450/KI21-1-5-3-2-2-30B-19-1) -2-2-1-1/SYN-Y-STR-34-1-1-1-1-2-1BB/KU1409xMO17LPAxKU1409-27-3-1-1-15*9 |
| En54 | (IITATZI1394 /Mp715/(9450/CM116/9450)-3-3-1-2-1*7/9450/KI21-1-5-3-2-2/DE3/9450/KI21-1-5-3-2-2-30B-19-1) -2-2-1-2/SYN-Y-STR-34-1-1-1-1-2-1BB/KU1409xMO17LPAxKU1409-27-3-1-1-15*9 |
| En55 | (IITATZI1394 /Mp715/(9450/CM116/9450)-3-3-1-2-1*7/9450/KI21-1-5-3-2-2/DE3/9450/KI21-1-5-3-2-2-30B-19-1) -5-2-1-1/SYN-Y-STR-34-1-1-1-1-2-1BB/KU1409xMO17LPAxKU1409-27-3-1-1-15*9 |
| En56 | (IITATZI1394 /Mp715/(9450/CM116/9450)-3-3-1-2-1*7/9450/KI21-1-5-3-2-2/DE3/9450/KI21-1-5-3-2-2-30B-19-1) -1-2-1-1-1/SYN-Y-STR-34-1-1-1-1-2-1BB/KU1409xMO17LPAxKU1409-27-3-1-1-15*9 |
| En57 | (IITATZI1394 /Mp715/(9450/CM116/9450)-3-3-1-2-1*7/9450/KI21-1-5-3-2-2/DE3/9450/KI21-1-5-3-2-2-30B-19-1) -1-2-1-1-2/SYN-Y-STR-34-1-1-1-1-2-1BB/KU1409xMO17LPAxKU1409-27-3-1-1-15*9 |
| En58 | (IITATZI1394 /Mp715/(9450/CM116/9450)-3-3-1-2-1*7/9450/KI21-1-5-3-2-2/DE3/9450/KI21-1-5-3-2-2-30B-19-1) -1-3-1-1-1/SYN-Y-STR-34-1-1-1-1-2-1BB/KU1409xMO17LPAxKU1409-27-3-1-1-15*9 |
| En59 | (IITATZI1394 /Mp715/(9450/CM116/9450)-3-3-1-2-1*7/9450/KI21-1-5-3-2-2/DE3/9450/KI21-1-5-3-2-2-30B-19-1) -1-3-1-1-2/SYN-Y-STR-34-1-1-1-1-2-1BB/KU1409xMO17LPAxKU1409-27-3-1-1-15*9 |
| En60 | (IITATZI1394 /Mp715/(9450/CM116/9450)-3-3-1-2-1*7/9450/KI21-1-5-3-2-2/DE3/9450/KI21-1-5-3-2-2-30B-19-1) -4-3-1-1/SYN-Y-STR-34-1-1-1-1-2-1BB/KU1409xMO17LPAxKU1409-27-3-1-1-15*9 |
| En61 | (IITATZI1394 /Mp715/(9450/CM116/9450)-3-3-1-2-1*7/9450/KI21-1-5-3-2-2/DE3/9450/KI21-1-5-3-2-2-30B-19-1) -4-3-1-2-1/SYN-Y-STR-34-1-1-1-1-2-1BB/KU1409xMO17LPAxKU1409-27-3-1-1-15*9 |
| En62 | (IITATZI1394 /Mp715/(9450/CM116/9450)-3-3-1-2-1*7/9450/KI21-1-5-3-2-2/DE3/9450/KI21-1-5-3-2-2-30B-19-1) -4-3-1-2-2/SYN-Y-STR-34-1-1-1-1-2-1BB/KU1409xMO17LPAxKU1409-27-3-1-1-15*9 |
| En63 | (IITATZI1394 /Mp715/(9450/CM116/9450)-3-3-1-2-1*7/9450/KI21-1-5-3-2-2/DE3/9450/KI21-1-5-3-2-2-30B-19-1) -4-3-1-4-1/SYN-Y-STR-34-1-1-1-1-2-1BB/KU1409xMO17LPAxKU1409-27-3-1-1-15*9 |
| En64 | (IITATZI1394 /Mp715/(9450/CM116/9450)-3-3-1-2-1*7/9450/KI21-1-5-3-2-2/DE3/9450/KI21-1-5-3-2-2-30B-19-1) -4-3-1-4-2/SYN-Y-STR-34-1-1-1-1-2-1BB/KU1409xMO17LPAxKU1409-27-3-1-1-15*9 |
| En65 | (IITATZI1394 /Mp715/(9450/CM116/9450)-3-3-1-2-1*7/9450/KI21-1-5-3-2-2/DE3/9450/KI21-1-5-3-2-2-30B-19-1) -1-1-1-1/SYN-Y-STR-34-1-1-1-1-2-1BB/KU1409xMO17LPAxKU1409-27-3-1-1-15*9 |
| En66 | (IITATZI1394 /Mp715/(9450/CM116/9450)-3-3-1-2-1*7/9450/KI21-1-5-3-2-2/DE3/9450/KI21-1-5-3-2-2-30B-19-1) -2-4-1-1/SYN-Y-STR-34-1-1-1-1-2-1BB/KU1409xMO17LPAxKU1409-27-3-1-1-15*9 |
| En67 | (IITATZI1394 /Mp715/(9450/CM116/9450)-3-3-1-2-1*7/9450/KI21-1-5-3-2-2/DE3/9450/KI21-1-5-3-2-2-30B-19-1) -1-1-1-1-1/SYN-Y-STR-34-1-1-1-1-2-1BB/KU1409xMO17LPAxKU1409-27-3-1-1-15*9 |
| En68 | (IITATZI1394 /Mp715/(9450/CM116/9450)-3-3-1-2-1*7/9450/KI21-1-5-3-2-2/DE3/9450/KI21-1-5-3-2-2-30B-19-1) -1-1-1-1-2/SYN-Y-STR-34-1-1-1-1-2-1BB/KU1409xMO17LPAxKU1409-27-3-1-1-15*9 |
| En69 | (IITATZI1394 /Mp715/(9450/CM116/9450)-3-3-1-2-1*7/9450/KI21-1-5-3-2-2/DE3/9450/KI21-1-5-3-2-2-30B-19-1) -4-2-1-1/SYN-Y-STR-34-1-1-1-1-2-1BB/KU1409xMO17LPAxKU1409-27-3-1-1-15*9 |
| En70 | (IITATZI1394 /Mp715/(9450/CM116/9450)-3-3-1-2-1*7/9450/KI21-1-5-3-2-2/DE3/9450/KI21-1-5-3-2-2-30B-19-1) -4-2-1-2/SYN-Y-STR-34-1-1-1-1-2-1BB/KU1409xMO17LPAxKU1409-27-3-1-1-15*9 |
| En71 | (IITATZI1394 /Mp715/(9450/CM116/9450)-3-3-1-2-1*7/9450/KI21-1-5-3-2-2/DE3/9450/KI21-1-5-3-2-2-30B-19-1) -5-1-1-1-1/SYN-Y-STR-34-1-1-1-1-2-1BB/KU1409xMO17LPAxKU1409-27-3-1-1-15*9 |
| En72 | (IITATZI1394 /Mp715/(9450/CM116/9450)-3-3-1-2-1*7/9450/KI21-1-5-3-2-2/DE3/9450/KI21-1-5-3-2-2-30B-19-1) -5-1-1-1-2/SYN-Y-STR-34-1-1-1-1-2-1BB/KU1409xMO17LPAxKU1409-27-3-1-1-15*9 |
| En73 | (IITATZI1394 /Mp715/(9450/CM116/9450)-3-3-1-2-1*7/9450/KI21-1-5-3-2-2/DE3/9450/KI21-1-5-3-2-2-30B-19-1) -4-3-1-4-1/SYN-Y-STR-34-1-1-1-1-2-1BB/KU1409xMO17LPAxKU1409-27-3-1-1-15*9 |
| En74 | (IITATZI1394 /Mp715/(9450/CM116/9450)-3-3-1-2-1*7/9450/KI21-1-5-3-2-2/DE3/9450/KI21-1-5-3-2-2-30B-19-1) -4-3-1-4-2/SYN-Y-STR-34-1-1-1-1-2-1BB/KU1409xMO17LPAxKU1409-27-3-1-1-15*9 |
| En75 | (IITATZI1394 /Mp715/(9450/CM116/9450)-3-3-1-2-1*7/9450/KI21-1-5-3-2-2/DE3/9450/KI21-1-5-3-2-2-30B-19-1) -3-2-3-1-1/SYN-Y-STR-34-1-1-1-1-2-1BB/KU1409xMO17LPAxKU1409-27-3-1-1-15*9 |
| En76 | (IITATZI1394 /Mp715/(9450/CM116/9450)-3-3-1-2-1*7/9450/KI21-1-5-3-2-2/DE3/9450/KI21-1-5-3-2-2-30B-19-1) -3-2-3-1-2/SYN-Y-STR-34-1-1-1-1-2-1BB/KU1409xMO17LPAxKU1409-27-3-1-1-15*9 |
| En77 | (IITATZI1394 /Mp715/(9450/CM116/9450)-3-3-1-2-1*7/9450/KI21-1-5-3-2-2/DE3/9450/KI21-1-5-3-2-2-30B-19-1) -3-2-3-1-1/SYN-Y-STR-34-1-1-1-1-2-1BB/KU1409xMO17LPAxKU1409-27-3-1-1-15*9 |
| En78 | (IITATZI1394 /Mp715/(9450/CM116/9450)-3-3-1-2-1*7/9450/KI21-1-5-3-2-2/DE3/9450/KI21-1-5-3-2-2-30B-19-1) -3-2-3-1-2/SYN-Y-STR-34-1-1-1-1-2-1BB/KU1409xMO17LPAxKU1409-27-3-1-1-15*9 |
| En79 | (IITATZI1394 /Mp715/(9450/CM116/9450)-3-3-1-2-1*7/9450/KI21-1-5-3-2-2/DE3/9450/KI21-1-5-3-2-2-30B-19-1) -1-1-2-1/SYN-Y-STR-34-1-1-1-1-2-1BB/KU1409xMO17LPAxKU1409-27-3-1-1-15*9 |
| En80 | (IITATZI1394 /Mp715/(9450/CM116/9450)-3-3-1-2-1*7/9450/KI21-1-5-3-2-2/DE3/9450/KI21-1-5-3-2-2-30B-19-1) -5-1-1-1-1/SYN-Y-STR-34-1-1-1-1-2-1BB/KU1409xMO17LPAxKU1409-27-3-1-1-15*9 |
| En81 | (IITATZI1394 /Mp715/(9450/CM116/9450)-3-3-1-2-1*7/9450/KI21-1-5-3-2-2/DE3/9450/KI21-1-5-3-2-2-30B-19-1) -5-1-1-1-2/SYN-Y-STR-34-1-1-1-1-2-1BB/KU1409xMO17LPAxKU1409-27-3-1-1-15*9 |
| En82 | (IITATZI1394 /Mp715/(9450/CM116/9450)-3-3-1-2-1*7/9450/KI21-1-5-3-2-2/DE3/9450/KI21-1-5-3-2-2-30B-19-1) -5-1-3-1/SYN-Y-STR-34-1-1-1-1-2-1BB/KU1409xMO17LPAxKU1409-27-3-1-1-15*9 |
| En83 | (IITATZI1394 /Mp715/(9450/CM116/9450)-3-3-1-2-1*7/9450/KI21-1-5-3-2-2/DE3/9450/KI21-1-5-3-2-2-30B-19-1) -2-1-1-1/SYN-Y-STR-34-1-1-1-1-2-1BB/KU1409xMO17LPAxKU1409-27-3-1-1-15*9 |
| En84 | (IITATZI1394 /Mp715/(9450/CM116/9450)-3-3-1-2-1*7/9450/KI21-1-5-3-2-2/DE3/9450/KI21-1-5-3-2-2-30B-19-1) -1-2-1/(ACR97TZL-CCOMP1-Y-S3-13-1B/CI7/ACR97TZL-CCOMP1-Y-S3-13-1B)-8*5 |
| En85 | (IITATZI1394 /Mp715/(9450/CM116/9450)-3-3-1-2-1*7/9450/KI21-1-5-3-2-2/DE3/9450/KI21-1-5-3-2-2-30B-19-1) -4-3-1/(ACR97TZL-CCOMP1-Y-S3-13-1B/CI7/ACR97TZL-CCOMP1-Y-S3-13-1B)-8*5 |
| En86 | (IITATZI1394 /Mp715/(9450/CM116/9450)-3-3-1-2-1*7/9450/KI21-1-5-3-2-2/DE3/9450/KI21-1-5-3-2-2-30B-19-1) -6-2-1-1-2-1/(ACR97TZL-CCOMP1-Y-S3-13-1B/CI7/ACR97TZL-CCOMP1-Y-S3-13-1B)-8*5 |
| En87 | (IITATZI1394 /Mp715/(9450/CM116/9450)-3-3-1-2-1*7/9450/KI21-1-5-3-2-2/DE3/9450/KI21-1-5-3-2-2-30B-19-1) -6-2-1-1-2-2/(ACR97TZL-CCOMP1-Y-S3-13-1B/CI7/ACR97TZL-CCOMP1-Y-S3-13-1B)-8*5 |
| En88 | (IITATZI1394 /Mp715/(9450/CM116/9450)-3-3-1-2-1*7/9450/KI21-1-5-3-2-2/DE3/9450/KI21-1-5-3-2-2-30B-19-1) -6-4-1/(ACR97TZL-CCOMP1-Y-S3-13-1B/CI7/ACR97TZL-CCOMP1-Y-S3-13-1B)-8*5 |
| En89 | (IITATZI1394 /Mp715/(9450/CM116/9450)-3-3-1-2-1*7/9450/KI21-1-5-3-2-2/DE3/9450/KI21-1-5-3-2-2-30B-19-1) -6-4-2-1-1/(ACR97TZL-CCOMP1-Y-S3-13-1B/CI7/ACR97TZL-CCOMP1-Y-S3-13-1B)-8*5 |
| En90 | (IITATZI1394 /Mp715/(9450/CM116/9450)-3-3-1-2-1*7/9450/KI21-1-5-3-2-2/DE3/9450/KI21-1-5-3-2-2-30B-19-1) -5-3-2/(ACR97TZL-CCOMP1-Y-S3-13-1B/CI7/ACR97TZL-CCOMP1-Y-S3-13-1B)-8*5 |
| En91 | (IITATZI1394 /Mp715/(9450/CM116/9450)-3-3-1-2-1*7/9450/KI21-1-5-3-2-2/DE3/9450/KI21-1-5-3-2-2-30B-19-1) -1-1-1/(ACR97TZL-CCOMP1-Y-S3-13-1B/CI7/ACR97TZL-CCOMP1-Y-S3-13-1B)-8*5 |
| En92 | (IITATZI1394 /Mp715/(9450/CM116/9450)-3-3-1-2-1*7/9450/KI21-1-5-3-2-2/DE3/9450/KI21-1-5-3-2-2-30B-19-1) -2-1-1-1/(ACR97TZL-CCOMP1-Y-S3-13-1B/CI7/ACR97TZL-CCOMP1-Y-S3-13-1B)-8*5 |
| En93 | (IITATZI1394 /Mp715/(9450/CM116/9450)-3-3-1-2-1*7/9450/KI21-1-5-3-2-2/DE3/9450/KI21-1-5-3-2-2-30B-19-1) -2-1-2-1-1/(ACR97TZL-CCOMP1-Y-S3-13-1B/CI7/ACR97TZL-CCOMP1-Y-S3-13-1B)-8*5 |
| En94 | (IITATZI1394 /Mp715/(9450/CM116/9450)-3-3-1-2-1*7/9450/KI21-1-5-3-2-2/DE3/9450/KI21-1-5-3-2-2-30B-19-1) -2-1-2-1-2/(ACR97TZL-CCOMP1-Y-S3-13-1B/CI7/ACR97TZL-CCOMP1-Y-S3-13-1B)-8*5 |
| En95 | (IITATZI1394 /Mp715/(9450/CM116/9450)-3-3-1-2-1*7/9450/KI21-1-5-3-2-2/DE3/9450/KI21-1-5-3-2-2-30B-19-1) -2-1-2-1-3/(ACR97TZL-CCOMP1-Y-S3-13-1B/CI7/ACR97TZL-CCOMP1-Y-S3-13-1B)-8*5 |
| En96 | (IITATZI1394 /Mp715/(9450/CM116/9450)-3-3-1-2-1*7/9450/KI21-1-5-3-2-2/DE3/9450/KI21-1-5-3-2-2-30B-19-1) -1-1-2-1-1/(ACR97TZL-CCOMP1-Y-S3-13-1B/CI7/ACR97TZL-CCOMP1-Y-S3-13-1B)-8*5 |
| En97 | (IITATZI1394 /Mp715/(9450/CM116/9450)-3-3-1-2-1*7/9450/KI21-1-5-3-2-2/DE3/9450/KI21-1-5-3-2-2-30B-19-1) -1-1-2-1-2/(ACR97TZL-CCOMP1-Y-S3-13-1B/CI7/ACR97TZL-CCOMP1-Y-S3-13-1B)-8*5 |
| En98 | (IITATZI1394 /Mp715/(9450/CM116/9450)-3-3-1-2-1*7/9450/KI21-1-5-3-2-2/DE3/9450/KI21-1-5-3-2-2-30B-19-1) -1-1-2-2/(ACR97TZL-CCOMP1-Y-S3-13-1B/CI7/ACR97TZL-CCOMP1-Y-S3-13-1B)-8*5 |
| En99 | (IITATZI1394 /Mp715/(9450/CM116/9450)-3-3-1-2-1*7/9450/KI21-1-5-3-2-2/DE3/9450/KI21-1-5-3-2-2-30B-19-1) -1-1-2-3/(ACR97TZL-CCOMP1-Y-S3-13-1B/CI7/ACR97TZL-CCOMP1-Y-S3-13-1B)-8*5 |
| En100 | (IITATZI1394 /Mp715/(9450/CM116/9450)-3-3-1-2-1*7/9450/KI21-1-5-3-2-2/DE3/9450/KI21-1-5-3-2-2-30B-19-1) -1-1-1-3/(ACR97TZL-CCOMP1-Y-S3-13-1B/CI7/ACR97TZL-CCOMP1-Y-S3-13-1B)-8*5 |
| En101 | (IITATZI1394 /Mp715/(9450/CM116/9450)-3-3-1-2-1*7/9450/KI21-1-5-3-2-2/DE3/9450/KI21-1-5-3-2-2-30B-19-1) -1-1-1-4/(ACR97TZL-CCOMP1-Y-S3-13-1B/CI7/ACR97TZL-CCOMP1-Y-S3-13-1B)-8*5 |
| En102 | (IITATZI1394 /Mp715/(9450/CM116/9450)-3-3-1-2-1*7/9450/KI21-1-5-3-2-2/DE3/9450/KI21-1-5-3-2-2-30B-19-1) -1-1-2-1/(ACR97TZL-CCOMP1-Y-S3-13-1B/CI7/ACR97TZL-CCOMP1-Y-S3-13-1B)-8*5 |
| En103 | (IITATZI1394 /Mp715/(9450/CM116/9450)-3-3-1-2-1*7/9450/KI21-1-5-3-2-2/DE3/9450/KI21-1-5-3-2-2-30B-19-1) -2-2-1-1/(ACR97TZL-CCOMP1-Y-S3-13-1B/CI7/ACR97TZL-CCOMP1-Y-S3-13-1B)-8*5 |
| En104 | (IITATZI1394 /Mp715/(9450/CM116/9450)-3-3-1-2-1*7/9450/KI21-1-5-3-2-2/DE3/9450/KI21-1-5-3-2-2-30B-19-1) -5-1-1-2/(ACR97TZL-CCOMP1-Y-S3-13-1B/CI7/ACR97TZL-CCOMP1-Y-S3-13-1B)-8*5 |
| En105 | (IITATZI1394 /Mp715/(9450/CM116/9450)-3-3-1-2-1*7/9450/KI21-1-5-3-2-2/DE3/9450/KI21-1-5-3-2-2-30B-19-1) -6-5-1-1/(ACR97TZL-CCOMP1-Y-S3-13-1B/CI7/ACR97TZL-CCOMP1-Y-S3-13-1B)-8*5 |
| En106 | (IITATZI1394 /Mp715/(9450/CM116/9450)-3-3-1-2-1*7/9450/KI21-1-5-3-2-2/DE3/9450/KI21-1-5-3-2-2-30B-19-1) -1-1-1-2/(ACR97TZL-CCOMP1-Y-S3-13-1B/CI7/ACR97TZL-CCOMP1-Y-S3-13-1B)-8*5 |
| En107 | (IITATZI1394 /Mp715/(9450/CM116/9450)-3-3-1-2-1*7/9450/KI21-1-5-3-2-2/DE3/9450/KI21-1-5-3-2-2-30B-19-1) -7-1-1-2/(ACR97TZL-CCOMP1-Y-S3-13-1B/CI7/ACR97TZL-CCOMP1-Y-S3-13-1B)-8*5 |
| En108 | (IITATZI1394 /Mp715/(9450/CM116/9450)-3-3-1-2-1*7/9450/KI21-1-5-3-2-2/DE3/9450/KI21-1-5-3-2-2-30B-19-1) -9-3-1-1/(ACR97TZL-CCOMP1-Y-S3-13-1B/CI7/ACR97TZL-CCOMP1-Y-S3-13-1B)-8*5 |
| En109 | (IITATZI1394 /Mp715/(9450/CM116/9450)-3-3-1-2-1*7/9450/KI21-1-5-3-2-2/DE3/9450/KI21-1-5-3-2-2-30B-19-1) -10-3-2-1/(ACR97TZL-CCOMP1-Y-S3-13-1B/CI7/ACR97TZL-CCOMP1-Y-S3-13-1B)-8*5 |
| En110 | (IITATZI1394 /Mp715/(9450/CM116/9450)-3-3-1-2-1*7/9450/KI21-1-5-3-2-2/DE3/9450/KI21-1-5-3-2-2-30B-19-1) -10-3-3-2/(ACR97TZL-CCOMP1-Y-S3-13-1B/CI7/ACR97TZL-CCOMP1-Y-S3-13-1B)-8*5 |
| En111 | (IITATZI1394 /Mp715/(9450/CM116/9450)-3-3-1-2-1*7/9450/KI21-1-5-3-2-2/DE3/9450/KI21-1-5-3-2-2-30B-19-1) -1-1-3-1/(ACR97TZL-CCOMP1-Y-S3-13-1B/CI7/ACR97TZL-CCOMP1-Y-S3-13-1B)-8*5 |
| En112 | (IITATZI1394 /Mp715/(9450/CM116/9450)-3-3-1-2-1*7/9450/KI21-1-5-3-2-2/DE3/9450/KI21-1-5-3-2-2-30B-19-1) -2-2-1-1/(ACR97TZL-CCOMP1-Y-S3-13-1B/CI7/ACR97TZL-CCOMP1-Y-S3-13-1B)-8*5 |
| En113 | (IITATZI1394 /Mp715/(9450/CM116/9450)-3-3-1-2-1*7/9450/KI21-1-5-3-2-2/DE3/9450/KI21-1-5-3-2-2-30B-19-1) -6-1-2-1/(ACR97TZL-CCOMP1-Y-S3-13-1B/CI7/ACR97TZL-CCOMP1-Y-S3-13-1B)-8*5 |
| En114 | (IITATZI1394 /Mp715/(9450/CM116/9450)-3-3-1-2-1*7/9450/KI21-1-5-3-2-2/DE3/9450/KI21-1-5-3-2-2-30B-19-1) -6-1-2-2/(ACR97TZL-CCOMP1-Y-S3-13-1B/CI7/ACR97TZL-CCOMP1-Y-S3-13-1B)-8*5 |
| En115 | (IITATZI1394 /Mp715/(9450/CM116/9450)-3-3-1-2-1*7/9450/KI21-1-5-3-2-2/DE3/9450/KI21-1-5-3-2-2-30B-19-1) -3-1-1-1/(ACR97TZL-CCOMP1-Y-S3-13-1B/CI7/ACR97TZL-CCOMP1-Y-S3-13-1B)-8*5 |
| En116 | (IITATZI1394 /Mp715/(9450/CM116/9450)-3-3-1-2-1*7/9450/KI21-1-5-3-2-2/DE3/9450/KI21-1-5-3-2-2-30B-19-1) -3-1-1-2/(ACR97TZL-CCOMP1-Y-S3-13-1B/CI7/ACR97TZL-CCOMP1-Y-S3-13-1B)-8*5 |
| En117 | (IITATZI1394 /Mp715/(9450/CM116/9450)-3-3-1-2-1*7/9450/KI21-1-5-3-2-2/DE3/9450/KI21-1-5-3-2-2-30B-19-1) -2-4-1-2/(ACR97TZL-CCOMP1-Y-S3-13-1B/CI7/ACR97TZL-CCOMP1-Y-S3-13-1B)-8*5 |
| En118 | (IITATZI1394 /Mp715/(9450/CM116/9450)-3-3-1-2-1*7/9450/KI21-1-5-3-2-2/DE3/9450/KI21-1-5-3-2-2-30B-19-1) -2-1-2-1/(ACR97TZL-CCOMP1-Y-S3-13-1B/CI7/ACR97TZL-CCOMP1-Y-S3-13-1B)-8*5 |
| En119 | (IITATZI1394 /Mp715/(9450/CM116/9450)-3-3-1-2-1*7/9450/KI21-1-5-3-2-2/DE3/9450/KI21-1-5-3-2-2-30B-19-1) -2-2-1-1/(ACR97TZL-CCOMP1-Y-S3-13-1B/CI7/ACR97TZL-CCOMP1-Y-S3-13-1B)-8*5 |
| En120 | (IITATZI1394 /Mp715/(9450/CM116/9450)-3-3-1-2-1*7/9450/KI21-1-5-3-2-2/DE3/9450/KI21-1-5-3-2-2-30B-19-1) -2-2-1-2-1/(ACR97TZL-CCOMP1-Y-S3-13-1B/CI7/ACR97TZL-CCOMP1-Y-S3-13-1B)-8*5 |
| En121 | (IITATZI1394 /Mp715/(9450/CM116/9450)-3-3-1-2-1*7/9450/KI21-1-5-3-2-2/DE3/9450/KI21-1-5-3-2-2-30B-19-1) -2-2-1-2-2/(ACR97TZL-CCOMP1-Y-S3-13-1B/CI7/ACR97TZL-CCOMP1-Y-S3-13-1B)-8*5 |
| En122 | (IITATZI1394 /Mp715/(9450/CM116/9450)-3-3-1-2-1*7/9450/KI21-1-5-3-2-2/DE3/9450/KI21-1-5-3-2-2-30B-19-1) -2-2-1-1/(ACR97TZL-CCOMP1-Y-S3-13-1B/CI7/ACR97TZL-CCOMP1-Y-S3-13-1B)-8*5 |
| En123 | (IITATZI1394 /Mp715/(9450/CM116/9450)-3-3-1-2-1*7/9450/KI21-1-5-3-2-2/DE3/9450/KI21-1-5-3-2-2-30B-19-1) -4-2-1-1/(ACR97TZL-CCOMP1-Y-S3-13-1B/CI7/ACR97TZL-CCOMP1-Y-S3-13-1B)-8*5 |
| En124 | (IITATZI1394 /Mp715/(9450/CM116/9450)-3-3-1-2-1*7/9450/KI21-1-5-3-2-2/DE3/9450/KI21-1-5-3-2-2-30B-19-1) -1-2-1-1-1/(ACR97TZL-CCOMP1-Y-S3-13-1B/CI7/ACR97TZL-CCOMP1-Y-S3-13-1B)-8*5 |
| En125 | (IITATZI1394 /Mp715/(9450/CM116/9450)-3-3-1-2-1*7/9450/KI21-1-5-3-2-2/DE3/9450/KI21-1-5-3-2-2-30B-19-1) -1-2-1-1-2/(ACR97TZL-CCOMP1-Y-S3-13-1B/CI7/ACR97TZL-CCOMP1-Y-S3-13-1B)-8*5 |
| En126 | (IITATZI1394 /Mp715/(9450/CM116/9450)-3-3-1-2-1*7/9450/KI21-1-5-3-2-2/DE3/9450/KI21-1-5-3-2-2-30B-19-1) -1-1-1-1-1/(ACR97TZL-CCOMP1-Y-S3-13-1B/CI7/ACR97TZL-CCOMP1-Y-S3-13-1B)-8*5 |
| En127 | (IITATZI1394 /Mp715/(9450/CM116/9450)-3-3-1-2-1*7/9450/KI21-1-5-3-2-2/DE3/9450/KI21-1-5-3-2-2-30B-19-1) -1-1-1-1-2/(ACR97TZL-CCOMP1-Y-S3-13-1B/CI7/ACR97TZL-CCOMP1-Y-S3-13-1B)-8*5 |
| En128 | (IITATZI1394 /Mp715/(9450/CM116/9450)-3-3-1-2-1*7/9450/KI21-1-5-3-2-2/DE3/9450/KI21-1-5-3-2-2-30B-19-1) -2-2-1-2-1/(ACR97TZL-CCOMP1-Y-S3-13-1B/CI7/ACR97TZL-CCOMP1-Y-S3-13-1B)-8*5 |
| En129 | (IITATZI1394 /Mp715/(9450/CM116/9450)-3-3-1-2-1*7/9450/KI21-1-5-3-2-2/DE3/9450/KI21-1-5-3-2-2-30B-19-1) -2-2-1-2-2/(ACR97TZL-CCOMP1-Y-S3-13-1B/CI7/ACR97TZL-CCOMP1-Y-S3-13-1B)-8*5 |
| En130 | (IITATZI1394 /Mp715/(9450/CM116/9450)-3-3-1-2-1*7/9450/KI21-1-5-3-2-2/DE3/9450/KI21-1-5-3-2-2-30B-19-1) -2-1-2-1/(ACR97TZL-CCOMP1-Y-S3-13-1B/CI7/ACR97TZL-CCOMP1-Y-S3-13-1B)-8*5 |
| En131 | (IITATZI1394 /Mp715/(9450/CM116/9450)-3-3-1-2-1*7/9450/KI21-1-5-3-2-2/DE3/9450/KI21-1-5-3-2-2-30B-19-1) -2-1-2-2/(ACR97TZL-CCOMP1-Y-S3-13-1B/CI7/ACR97TZL-CCOMP1-Y-S3-13-1B)-8*5 |
| En132 | (IITATZI1394 /Mp715/(9450/CM116/9450)-3-3-1-2-1*7/9450/KI21-1-5-3-2-2/DE3/9450/KI21-1-5-3-2-2-30B-19-1) -2-1-2-3-1/(ACR97TZL-CCOMP1-Y-S3-13-1B/CI7/ACR97TZL-CCOMP1-Y-S3-13-1B)-8*5 |
| En133 | (IITATZI1394 /Mp715/(9450/CM116/9450)-3-3-1-2-1*7/9450/KI21-1-5-3-2-2/DE3/9450/KI21-1-5-3-2-2-30B-19-1) -2-1-2-3-2/(ACR97TZL-CCOMP1-Y-S3-13-1B/CI7/ACR97TZL-CCOMP1-Y-S3-13-1B)-8*5 |
| En134 | (IITATZI1394 /Mp715/(9450/CM116/9450)-3-3-1-2-1*7/9450/KI21-1-5-3-2-2/DE3/9450/KI21-1-5-3-2-2-30B-19-1) -4-1-1-3/(ACR97TZL-CCOMP1-Y-S3-13-1B/CI7/ACR97TZL-CCOMP1-Y-S3-13-1B)-8*5 |
| En135 | (IITATZI1394 /Mp715/(9450/CM116/9450)-3-3-1-2-1*7/9450/KI21-1-5-3-2-2/DE3/9450/KI21-1-5-3-2-2-30B-19-1) -5-2-1-2/(ACR97TZL-CCOMP1-Y-S3-13-1B/CI7/ACR97TZL-CCOMP1-Y-S3-13-1B)-8*5 |
| En136 | (IITATZI1394 /Mp715/(9450/CM116/9450)-3-3-1-2-1*7/9450/KI21-1-5-3-2-2/DE3/9450/KI21-1-5-3-2-2-30B-19-1) -2-2-1-1/(ACR97TZL-CCOMP1-Y-S3-13-1B/CI7/ACR97TZL-CCOMP1-Y-S3-13-1B)-8*5 |
| En137 | (IITATZI1394 /Mp715/(9450/CM116/9450)-3-3-1-2-1*7/9450/KI21-1-5-3-2-2/DE3/9450/KI21-1-5-3-2-2-30B-19-1) -2-2-1-2/(ACR97TZL-CCOMP1-Y-S3-13-1B/CI7/ACR97TZL-CCOMP1-Y-S3-13-1B)-8*5 |
| En138 | (IITATZI1394 /Mp715/(9450/CM116/9450)-3-3-1-2-1*7/9450/KI21-1-5-3-2-2/DE3/9450/KI21-1-5-3-2-2-30B-19-1) -5-2-1-1/(ACR97TZL-CCOMP1-Y-S3-13-1B/CI7/ACR97TZL-CCOMP1-Y-S3-13-1B)-8*5 |
| En139 | (IITATZI1394 /Mp715/(9450/CM116/9450)-3-3-1-2-1*7/9450/KI21-1-5-3-2-2/DE3/9450/KI21-1-5-3-2-2-30B-19-1) -1-2-1-1-1/(ACR97TZL-CCOMP1-Y-S3-13-1B/CI7/ACR97TZL-CCOMP1-Y-S3-13-1B)-8*5 |
| En140 | (IITATZI1394 /Mp715/(9450/CM116/9450)-3-3-1-2-1*7/9450/KI21-1-5-3-2-2/DE3/9450/KI21-1-5-3-2-2-30B-19-1) -1-2-1-1-2/(ACR97TZL-CCOMP1-Y-S3-13-1B/CI7/ACR97TZL-CCOMP1-Y-S3-13-1B)-8*5 |
| En141 | (IITATZI1394 /Mp715/(9450/CM116/9450)-3-3-1-2-1*7/9450/KI21-1-5-3-2-2/DE3/9450/KI21-1-5-3-2-2-30B-19-1) -1-3-1-1-1/(ACR97TZL-CCOMP1-Y-S3-13-1B/CI7/ACR97TZL-CCOMP1-Y-S3-13-1B)-8*5 |
| En142 | (IITATZI1394 /Mp715/(9450/CM116/9450)-3-3-1-2-1*7/9450/KI21-1-5-3-2-2/DE3/9450/KI21-1-5-3-2-2-30B-19-1) -1-3-1-1-2/(ACR97TZL-CCOMP1-Y-S3-13-1B/CI7/ACR97TZL-CCOMP1-Y-S3-13-1B)-8*5 |
| En143 | (IITATZI1394 /Mp715/(9450/CM116/9450)-3-3-1-2-1*7/9450/KI21-1-5-3-2-2/DE3/9450/KI21-1-5-3-2-2-30B-19-1) -4-3-1-1/(ACR97TZL-CCOMP1-Y-S3-13-1B/CI7/ACR97TZL-CCOMP1-Y-S3-13-1B)-8*5 |
| En144 | (IITATZI1394 /Mp715/(9450/CM116/9450)-3-3-1-2-1*7/9450/KI21-1-5-3-2-2/DE3/9450/KI21-1-5-3-2-2-30B-19-1) -4-3-1-2-1/(ACR97TZL-CCOMP1-Y-S3-13-1B/CI7/ACR97TZL-CCOMP1-Y-S3-13-1B)-8*5 |
| En145 | (IITATZI1394 /Mp715/(9450/CM116/9450)-3-3-1-2-1*7/9450/KI21-1-5-3-2-2/DE3/9450/KI21-1-5-3-2-2-30B-19-1) -4-3-1-2-2/(ACR97TZL-CCOMP1-Y-S3-13-1B/CI7/ACR97TZL-CCOMP1-Y-S3-13-1B)-8*5 |
| En146 | (IITATZI1394 /Mp715/(9450/CM116/9450)-3-3-1-2-1*7/9450/KI21-1-5-3-2-2/DE3/9450/KI21-1-5-3-2-2-30B-19-1) -4-3-1-4-1/(ACR97TZL-CCOMP1-Y-S3-13-1B/CI7/ACR97TZL-CCOMP1-Y-S3-13-1B)-8*5 |
| En147 | (IITATZI1394 /Mp715/(9450/CM116/9450)-3-3-1-2-1*7/9450/KI21-1-5-3-2-2/DE3/9450/KI21-1-5-3-2-2-30B-19-1) -4-3-1-4-2/(ACR97TZL-CCOMP1-Y-S3-13-1B/CI7/ACR97TZL-CCOMP1-Y-S3-13-1B)-8*5 |
| En148 | (IITATZI1394 /Mp715/(9450/CM116/9450)-3-3-1-2-1*7/9450/KI21-1-5-3-2-2/DE3/9450/KI21-1-5-3-2-2-30B-19-1) -1-1-1-1/(ACR97TZL-CCOMP1-Y-S3-13-1B/CI7/ACR97TZL-CCOMP1-Y-S3-13-1B)-8*5 |
| En149 | (IITATZI1394 /Mp715/(9450/CM116/9450)-3-3-1-2-1*7/9450/KI21-1-5-3-2-2/DE3/9450/KI21-1-5-3-2-2-30B-19-1) -2-4-1-1/(ACR97TZL-CCOMP1-Y-S3-13-1B/CI7/ACR97TZL-CCOMP1-Y-S3-13-1B)-8*5 |
| En150 | (IITATZI1394 /Mp715/(9450/CM116/9450)-3-3-1-2-1*7/9450/KI21-1-5-3-2-2/DE3/9450/KI21-1-5-3-2-2-30B-19-1) -1-1-1-1-1/(ACR97TZL-CCOMP1-Y-S3-13-1B/CI7/ACR97TZL-CCOMP1-Y-S3-13-1B)-8*5 |
| En151 | (IITATZI1394 /Mp715/(9450/CM116/9450)-3-3-1-2-1*7/9450/KI21-1-5-3-2-2/DE3/9450/KI21-1-5-3-2-2-30B-19-1) -1-1-1-1-2/(ACR97TZL-CCOMP1-Y-S3-13-1B/CI7/ACR97TZL-CCOMP1-Y-S3-13-1B)-8*5 |
| En152 | (IITATZI1394 /Mp715/(9450/CM116/9450)-3-3-1-2-1*7/9450/KI21-1-5-3-2-2/DE3/9450/KI21-1-5-3-2-2-30B-19-1) -4-2-1-1/(ACR97TZL-CCOMP1-Y-S3-13-1B/CI7/ACR97TZL-CCOMP1-Y-S3-13-1B)-8*5 |
| En153 | (IITATZI1394 /Mp715/(9450/CM116/9450)-3-3-1-2-1*7/9450/KI21-1-5-3-2-2/DE3/9450/KI21-1-5-3-2-2-30B-19-1) -4-2-1-2/(ACR97TZL-CCOMP1-Y-S3-13-1B/CI7/ACR97TZL-CCOMP1-Y-S3-13-1B)-8*5 |
| En154 | (IITATZI1394 /Mp715/(9450/CM116/9450)-3-3-1-2-1*7/9450/KI21-1-5-3-2-2/DE3/9450/KI21-1-5-3-2-2-30B-19-1) -5-1-1-1-1/(ACR97TZL-CCOMP1-Y-S3-13-1B/CI7/ACR97TZL-CCOMP1-Y-S3-13-1B)-8*5 |
| En155 | (IITATZI1394 /Mp715/(9450/CM116/9450)-3-3-1-2-1*7/9450/KI21-1-5-3-2-2/DE3/9450/KI21-1-5-3-2-2-30B-19-1) -5-1-1-1-2/(ACR97TZL-CCOMP1-Y-S3-13-1B/CI7/ACR97TZL-CCOMP1-Y-S3-13-1B)-8*5 |
| En156 | (IITATZI1394 /Mp715/(9450/CM116/9450)-3-3-1-2-1*7/9450/KI21-1-5-3-2-2/DE3/9450/KI21-1-5-3-2-2-30B-19-1) -4-3-1-4-1/(ACR97TZL-CCOMP1-Y-S3-13-1B/CI7/ACR97TZL-CCOMP1-Y-S3-13-1B)-8*5 |
| En157 | (IITATZI1394 /Mp715/(9450/CM116/9450)-3-3-1-2-1*7/9450/KI21-1-5-3-2-2/DE3/9450/KI21-1-5-3-2-2-30B-19-1) -4-3-1-4-2/(ACR97TZL-CCOMP1-Y-S3-13-1B/CI7/ACR97TZL-CCOMP1-Y-S3-13-1B)-8*5 |
| En158 | (IITATZI1394 /Mp715/(9450/CM116/9450)-3-3-1-2-1*7/9450/KI21-1-5-3-2-2/DE3/9450/KI21-1-5-3-2-2-30B-19-1) -3-2-3-1-1/(ACR97TZL-CCOMP1-Y-S3-13-1B/CI7/ACR97TZL-CCOMP1-Y-S3-13-1B)-8*5 |
| En159 | (IITATZI1394 /Mp715/(9450/CM116/9450)-3-3-1-2-1*7/9450/KI21-1-5-3-2-2/DE3/9450/KI21-1-5-3-2-2-30B-19-1) -3-2-3-1-2/(ACR97TZL-CCOMP1-Y-S3-13-1B/CI7/ACR97TZL-CCOMP1-Y-S3-13-1B)-8*5 |
| En160 | (IITATZI1394 /Mp715/(9450/CM116/9450)-3-3-1-2-1*7/9450/KI21-1-5-3-2-2/DE3/9450/KI21-1-5-3-2-2-30B-19-1) -3-2-3-1-1/(ACR97TZL-CCOMP1-Y-S3-13-1B/CI7/ACR97TZL-CCOMP1-Y-S3-13-1B)-8*5 |
| En161 | (IITATZI1394 /Mp715/(9450/CM116/9450)-3-3-1-2-1*7/9450/KI21-1-5-3-2-2/DE3/9450/KI21-1-5-3-2-2-30B-19-1) -3-2-3-1-2/(ACR97TZL-CCOMP1-Y-S3-13-1B/CI7/ACR97TZL-CCOMP1-Y-S3-13-1B)-8*5 |
| En162 | (IITATZI1394 /Mp715/(9450/CM116/9450)-3-3-1-2-1*7/9450/KI21-1-5-3-2-2/DE3/9450/KI21-1-5-3-2-2-30B-19-1) -1-1-2-1/(ACR97TZL-CCOMP1-Y-S3-13-1B/CI7/ACR97TZL-CCOMP1-Y-S3-13-1B)-8*5 |
| En163 | (IITATZI1394 /Mp715/(9450/CM116/9450)-3-3-1-2-1*7/9450/KI21-1-5-3-2-2/DE3/9450/KI21-1-5-3-2-2-30B-19-1) -5-1-1-1-1/(ACR97TZL-CCOMP1-Y-S3-13-1B/CI7/ACR97TZL-CCOMP1-Y-S3-13-1B)-8*5 |
| En164 | (IITATZI1394 /Mp715/(9450/CM116/9450)-3-3-1-2-1*7/9450/KI21-1-5-3-2-2/DE3/9450/KI21-1-5-3-2-2-30B-19-1) -5-1-1-1-2/(ACR97TZL-CCOMP1-Y-S3-13-1B/CI7/ACR97TZL-CCOMP1-Y-S3-13-1B)-8*5 |
| En165 | (IITATZI1394 /Mp715/(9450/CM116/9450)-3-3-1-2-1*7/9450/KI21-1-5-3-2-2/DE3/9450/KI21-1-5-3-2-2-30B-19-1) -5-1-3-1/(ACR97TZL-CCOMP1-Y-S3-13-1B/CI7/ACR97TZL-CCOMP1-Y-S3-13-1B)-8*5 |
| En166 | (IITATZI1394 /Mp715/(9450/CM116/9450)-3-3-1-2-1*7/9450/KI21-1-5-3-2-2/DE3/9450/KI21-1-5-3-2-2-30B-19-1) -2-1-1-1/(ACR97TZL-CCOMP1-Y-S3-13-1B/CI7/ACR97TZL-CCOMP1-Y-S3-13-1B)-8*5 |
|  | Genetic hybrid checks |
| En167 | (ACR97TZL-CCOMP1-Y-S3-13-1B/CI7/ACR97TZL-CCOMP1-Y-S3-13-1B)-8*5/SYN-Y-STR-34-1-1-1-1-2-1BB/KU1409xMO17LPAxKU1409-27-3-1-1-15*9 |
| En168 | 8425-8 |
| En169 | 8338-1 |
|  | Commercial hybrid checks |
| En170 | Ife hybrid 3 |
| En171 | Ife hybrid 4 |
| En172 | SC645 |

Supplementary Table 2. BLUP estimates and MGIDI-based ranking of 33 selected testcross hybrids and checks for grain yield, aflatoxin accumulation, and Aspergillus ear rot severity

| **Testcrosses** | **AFLT** | **Rank based on aflatoxin** | **AER** | **GY** | **Rank based on yield** | **DTA** | **DTS** | **ASI** | **EA** | **PH** | **EH** | **MGIDI** | **Rank based on MGIDI** |
| --- | --- | --- | --- | --- | --- | --- | --- | --- | --- | --- | --- | --- | --- |
| En80 | 211.57 | 11 | 2.20 | 7.83 | 3 | 55.21 | 58.02 | 2.99 | 2.68 | 241.29 | 96.63 | 3.01 | 1 |
| En118 | 235.11 | 23 | 2.35 | 7.13 | 21 | 53.49 | 56.67 | 3.15 | 2.77 | 253.56 | 93.77 | 3.38 | 2 |
| En79 | 191.95 | 6 | 2.31 | 6.72 | 30 | 53.75 | 56.62 | 2.99 | 2.64 | 235.92 | 88.77 | 3.55 | 3.5 |
| En11 | 212.50 | 12 | 2.34 | 7.19 | 15 | 54.99 | 57.73 | 2.94 | 2.71 | 241.90 | 94.82 | 3.55 | 3.5 |
| En77 | 204.18 | 8 | 2.27 | 7.28 | 12 | 55.33 | 58.24 | 3.05 | 2.71 | 251.07 | 96.87 | 3.59 | 5 |
| En9 | 184.14 | 3 | 2.51 | 7.16 | 17.5 | 55.59 | 58.41 | 3.00 | 2.66 | 246.59 | 99.79 | 4.00 | 6.5 |
| En69 | 257.78 | 31 | 2.28 | 6.83 | 29 | 53.69 | 56.66 | 3.05 | 2.83 | 236.37 | 89.57 | 4.00 | 6.5 |
| En74 | 174.26 | 1 | 2.45 | 7.88 | 2 | 56.04 | 58.93 | 3.05 | 2.69 | 246.35 | 95.52 | 4.01 | 8 |
| En70 | 261.51 | 32 | 2.34 | 7.27 | 13 | 54.92 | 58.32 | 3.27 | 2.67 | 244.93 | 93.41 | 4.03 | 9 |
| En107 | 214.95 | 16 | 2.27 | 6.90 | 27 | 52.22 | 55.85 | 3.36 | 2.85 | 240.80 | 85.17 | 4.11 | 10 |
| En37 | 191.08 | 5 | 2.25 | 7.11 | 22 | 55.52 | 58.41 | 3.04 | 2.69 | 242.91 | 93.04 | 4.13 | 11 |
| En7 | 206.25 | 9 | 2.27 | 6.66 | 31.5 | 54.96 | 57.71 | 2.94 | 2.86 | 247.00 | 98.07 | 4.16 | 12 |
| En102 | 237.11 | 24 | 2.52 | 7.79 | 4 | 53.50 | 56.73 | 3.19 | 2.89 | 253.39 | 95.45 | 4.17 | 13 |
| En81 | 195.23 | 7 | 2.17 | 7.15 | 19.5 | 55.65 | 58.38 | 2.95 | 2.82 | 245.51 | 98.23 | 4.20 | 14 |
| En153 | 212.71 | 14 | 2.36 | 7.16 | 17.5 | 54.19 | 57.43 | 3.20 | 2.86 | 242.77 | 89.48 | 4.25 | 15 |
| En156 | 208.49 | 10 | 2.39 | 7.25 | 14 | 55.28 | 58.06 | 2.99 | 2.88 | 248.10 | 91.78 | 4.27 | 16 |
| En157 | 242.41 | 27 | 2.40 | 7.67 | 6.5 | 56.21 | 58.82 | 2.90 | 2.78 | 249.04 | 96.77 | 4.32 | 17 |
| En65 | 234.99 | 22 | 2.52 | 7.96 | 1 | 55.29 | 58.54 | 3.25 | 2.76 | 250.92 | 98.94 | 4.35 | 18 |
| En30 | 214.68 | 15 | 2.46 | 7.04 | 24 | 54.24 | 57.78 | 3.32 | 2.79 | 247.71 | 92.11 | 4.37 | 19 |
| En137 | 212.59 | 13 | 2.41 | 7.17 | 16 | 54.62 | 57.67 | 3.10 | 2.93 | 246.62 | 93.11 | 4.43 | 20 |
| En15 | 243.35 | 28 | 2.46 | 7.40 | 10.5 | 54.77 | 58.20 | 3.31 | 2.77 | 253.97 | 100.14 | 4.48 | 21 |
| En141 | 216.99 | 18 | 2.48 | 7.62 | 8 | 55.02 | 58.01 | 3.09 | 2.92 | 243.16 | 90.76 | 4.53 | 22.5 |
| En73 | 241.21 | 26 | 2.49 | 7.67 | 6.5 | 56.18 | 59.03 | 3.00 | 2.73 | 247.81 | 98.19 | 4.53 | 22.5 |
| En105 | 256.86 | 29 | 2.35 | 6.92 | 26 | 54.32 | 57.38 | 3.10 | 2.93 | 245.33 | 92.85 | 4.55 | 24 |
| En8 | 183.47 | 2 | 2.57 | 6.93 | 25 | 54.44 | 57.41 | 3.05 | 2.81 | 241.02 | 85.70 | 4.58 | 25 |
| En152 | 238.06 | 25 | 2.45 | 7.05 | 23 | 53.53 | 56.79 | 3.20 | 2.86 | 237.37 | 84.95 | 4.62 | 26.5 |
| En136 | 232.75 | 20 | 2.40 | 7.40 | 10.5 | 54.78 | 57.73 | 3.05 | 3.00 | 244.14 | 91.18 | 4.62 | 26.5 |
| En71 | 269.47 | 33 | 2.61 | 7.69 | 5 | 54.89 | 57.85 | 3.05 | 2.80 | 243.06 | 93.79 | 4.66 | 28 |
| En112 | 215.47 | 17 | 2.49 | 6.62 | 33 | 53.78 | 56.37 | 2.84 | 2.98 | 239.96 | 86.41 | 4.69 | 29 |
| En51 | 257.03 | 30 | 2.45 | 6.66 | 31.5 | 54.91 | 58.09 | 3.16 | 2.72 | 242.23 | 94.31 | 4.70 | 30 |
| En58 | 188.11 | 4 | 2.29 | 7.43 | 9 | 56.07 | 59.18 | 3.15 | 2.76 | 244.60 | 91.38 | 4.72 | 31 |
| En110 | 230.25 | 19 | 2.37 | 7.15 | 19.5 | 55.15 | 58.18 | 3.10 | 2.91 | 243.36 | 91.97 | 4.75 | 32.5 |
| En161 | 233.88 | 21 | 2.37 | 6.87 | 28 | 54.70 | 57.77 | 3.11 | 2.97 | 249.66 | 95.36 | 4.75 | 32.5 |
|  |  |  |  |  |  |  |  |  |  |  |  |  |  |
| Genetic checks hybrids | | |  |  |  |  |  |  |  |  |  |  |  |
| T1×T2 | 180.27 | - | 2.29 | 6.83 | - | 55.70 | 58.21 | 2.84 | 2.74 | 251.62 | 104.36 | - | - |
| 8425-8 | 251.14 | - | 2.48 | 6.55 | - | 54.34 | 57.52 | 3.16 | 2.84 | 243.08 | 94.54 | - | - |
| 8338-1 | 218.20 | - | 2.41 | 6.79 | - | 56.75 | 60.00 | 3.26 | 2.83 | 253.09 | 96.00 | - | - |
| Mean | 216.54 |  | 2.39 | 6.72 |  | 55.60 | 58.58 | 3.09 | 2.80 | 249.26 | 98.30 |  |  |
|  |  |  |  |  |  |  |  |  |  |  |  |  |  |
| Commercial check hybrids | | |  |  |  |  |  |  |  |  |  |  |  |
| Ife hybrid 3 | 234.10 | - | 2.53 | 5.87 | - | 55.15 | 58.26 | 3.15 | 3.03 | 230.27 | 97.13 | - | - |
| Ife hybrid 4 | 180.27 | - | 2.01 | 8.41 | - | 56.15 | 59.21 | 3.15 | 2.56 | 256.61 | 106.36 | - | - |
| SC645 | 227.15 | - | 2.64 | 5.88 | - | 57.26 | 61.03 | 3.52 | 2.99 | 250.11 | 100.38 | - | - |
| Mean | 213.84 |  | 2.39 | 6.72 |  | 56.19 | 59.5 | 3.27 | 2.99 | 250.66 | 101.29 |  |  |

GY: Grain yield; AFLT: Aflatoxin accumulation (B1 + B2); AER: Aspergillus ear rot; EA: Ear aspect; ASI: Anthesis-silking interval; DTA: Days to anthesis; DTS: Days to silking; PH: Plant height; EH: Ear height; MGIDI: Multi-trait genotype-ideotype distance index.
